# Signs of positive selection of somatic mutations in human cancers detected by EST sequence analysis

**DOI:** 10.1186/1471-2407-6-36

**Published:** 2006-02-09

**Authors:** Vladimir N Babenko, Malay K Basu, Fyodor A Kondrashov, Igor B Rogozin, Eugene V Koonin

**Affiliations:** 1 National Center for Biotechnology Information, National Library of Medicine, National Institutes of Health, Bethesda MD, USA; 2 Section of Ecology, Behavior and Evolution, University of California San Diego, La Jolla, CA, USA

## Abstract

**Background:**

Carcinogenesis typically involves multiple somatic mutations in caretaker (DNA repair) and gatekeeper (tumor suppressors and oncogenes) genes. Analysis of mutation spectra of the tumor suppressor that is most commonly mutated in human cancers, p53, unexpectedly suggested that somatic evolution of the p53 gene during tumorigenesis is dominated by positive selection for gain of function. This conclusion is supported by accumulating experimental evidence of evolution of new functions of p53 in tumors. These findings prompted a genome-wide analysis of possible positive selection during tumor evolution.

**Methods:**

A comprehensive analysis of probable somatic mutations in the sequences of Expressed Sequence Tags (ESTs) from malignant tumors and normal tissues was performed in order to access the prevalence of positive selection in cancer evolution. For each EST, the numbers of synonymous and non-synonymous substitutions were calculated. In order to identify genes with a signature of positive selection in cancers, these numbers were compared to: i) expected numbers and ii) the numbers for the respective genes in the ESTs from normal  tissues.

**Results:**

We identified 112 genes with a signature of positive selection in cancers, i.e., a significantly elevated ratio of non-synonymous to synonymous substitutions, in tumors as compared to 37 such genes in an approximately equal-sized EST collection from normal tissues. A substantial fraction of the tumor-specific positive-selection candidates have experimentally demonstrated or strongly predicted links to cancer.

**Conclusion:**

The results of EST analysis should be interpreted with extreme caution given the noise introduced by sequencing errors and undetected polymorphisms. Furthermore, an inherent limitation of EST analysis is that multiple mutations amenable to statistical analysis can be detected only in relatively highly expressed genes. Nevertheless, the present results suggest that positive selection might affect a substantial number of genes during tumorigenic somatic evolution.

## Background

It is well established that most cancers are triggered by somatic or, less commonly, germline mutations in caretaker and gatekeeper genes [1-6]. The caretakers are broadly defined DNA repair genes that are responsible for maintenance of genome stability. Mutations in the caretaker genes, which are considered to be typical tumor suppressors, compromise genome stability and, more specifically, increase the probability of mutation in the gatekeepers which include both tumor suppressor genes and oncogenes [3,7]. Tumor suppressors are genes that control cell proliferation, in particular, by causing cell death in response to DNA damage; accordingly, mutational inactivation of tumor suppressors may cause transformation. In contrast, oncogenes are genes that, when mutated, acquire new functions promoting cell proliferation and, eventually, transformation [4].

Since the pioneering work of Theodore Boveri in the beginning of the 20^th ^century[8], tumorigenesis often has been viewed as a somatic version of Darwinian evolution [9-12]. This perspective implies positive selection of mutations that are beneficial from the standpoint of an individual cell, i.e., mutations that promote cell proliferation such as those activating the tumorigenic potential of oncogenes and those inactivating tumor suppressors. In the context of modern evolutionary synthesis, it is equally obvious that tumor evolution should involve substantial purifying selection against mutations impairing proliferation. Although the Darwinian view of tumorigenesis seems to be increasingly gaining foothold, the interplay of selective forces acting on mutations in specific genes is not understood in detail.

Altogether, mutations in more than 200 human genes have been implicated in cancer [13]. Currently, inactivation of tumor suppressors is considered to be the main driving force of tumorigenesis. The most prominent and best studied tumor suppressor is *p53*, a multifunctional transactivator of transcription and regulator of cell proliferation, programmed cell death, and repair [14-16]. The *p53 *gene is mutated in nearly 60% of human tumors. Many independent studies have shown that, in addition to its tumor suppressor properties, p53 may also behave as an oncogene[17]. Specifically, gain of new biochemical (e.g., transactivation of transcription of genes that are not affected by wild-type p53) and biological (e.g,, stimulation of cell proliferation) functions resulting from p53 mutations has been demonstrated [18-22]. Compelling evidence of p53 gain-of-function during tumorigenesis has been provided by recent reports on mouse models of Li-Fraumeni syndrome (LFS), a familial cancer predisposition syndrome caused by germline p53 mutations. These studies revealed substantial changes in the tumor spectra of mice carrying common p53 mutations, indicating that gain-of-function by p53 is important for tumorigenesis[23,24].

The conclusion that gain-of-function in p53 mutants is important for tumorigenesis is strongly supported by the results of bioinformatic analysis of the mutation spectra of the *p53 *gene [25,26]. These studies yielded three lines of evidence compatible with biologically relevant gain-of-function in p53 mutants in tumors:

i) somatic mutations of p53 detected in various cancers showed a highly significant excess of non-synonymous over synonymous substitutions, which is the signature of positive selection[27], ii) amino acid replacements caused by cancer-associated mutations clustered within evolutionarily conserved, functionally important regions of p53, and iii) mutational hotspots, the sites of frequent mutation which are subject to particularly strong positive selection, differed depending on the type of tumor, which suggests acquisition of distinct new functions by p53 in different tumors.

These observations prompted us to ask whether positive selection could also be detected in somatic mutants of other cancer-related genes in tumors. Genes evolving under positive selection during cancer progression could be viewed as candidate new oncogenes. To delineate the repertoire of such genes, we performed a genome-wide search for positive selection during cancer evolution by comparing the sequences of Expressed Sequence Tags (EST[28]) from tumors to the corresponding genomic sequences. The rationale of this analysis is to detect somatic mutations in ESTs and identify genes that show a significant excess of non-synonymous over synonymous substitutions in tumors. In principle, EST libraries provide ample material for analyzing somatic mutations in tumors and normal tissues. The problem with this approach is that differences between EST sequences and the sequences of the respective reference genes from the human genome may be caused by a variety of reasons other than somatic mutation including sequencing errors, incorrect assignment of an EST to a reference gene, and single-nucleotide polymorphisms (SNPs).

Several recent, large scale studies employed EST collections for detecting cancer-associated SNPs and cancer-specific alternative splice forms. In particular, Xu and Lee identified 316 human splice variant forms with a statistically significant cancer association; the structures of the most abundant of these were supported by sequences of the corresponding mRNAs isolated from tumors [29]. Another, larger-scale study by Gupta et al. reported 1120 tumor-specific splice isoforms with a high rate of validation by mRNA sequencing. However, when mRNA analysis was performed, the tissue specificity of many of these transcripts, particularly, those of low abundance, could not be confirmed[30]. A study by Brentani et al. took a different approach by using ESTs to identify SNPs in a predefined set of cancer-related genes; this resulted in the identification of 237 previously known and 505 new SNPs in these genes[31]. A comprehensive analysis by Qiu and coworkers involved cross-mapping of the EST database (dbEST) and the database SNP (dbSNP), yielding a statistically significant association with tumors for 4865 SNPs[32].

These studies emphasize the potential of EST analysis for detecting genomic and expression features associated with cancer. However, they are not particularly informative in terms of uncovering potential causative roles of individual genes in tumorigenesis. We were interested in mining dbEST for somatic mutations that could be positively selected in cancers, which would make the respective genes candidate oncogenes. The inherent problem of such analysis is distinguishing somatic mutations from sequencing errors and SNPs. However, the latter two sources of sequence variation are not expected to produce a signature of positive selection. Indeed, whatever biases are prevalent among sequencing errors, they would not effect the non-synonymous to synonymous substitutions. The issue with SNPs, obviously, is more complex. However, most if not all human SNPs appear to be either selectively neutral or slightly deleterious and do not show signs of frequent positive selection[33,34]. Accordingly, the signature of positive selection, namely, an elevated non-synonymous/synonymous substitution ratio [27,35], is expected to be detectable among somatic mutations even in the presence of some contamination by sequencing errors and SNPs.

With this premise, we partitioned the EST sequence libraries available through the dbEST database (NCBI, NIH, Bethesda) into those originating from tumors (hereinafter cancer ESTs) and those from normal tissues (normal ESTs), and identified genes with a significant excess of non-synonymous substitutions in each of the two sets. The results suggest that positive selection is more pronounced in somatic evolution of tumors than it is in normal tissues. Many genes with a signature of positive selection in tumors have established or strongly predicted links to cancer.

## Results

### Signatures of purifying and positive selection in cancer ests

The ESTs from both tumors and normal cells showed a much lower ratio of non-synonymous to synonymous substitutions than expected under the model of neutral evolution (Table 1), indicating that most of these substitution were genuine mutations subject to purifying selection rather than sequencing errors or neutral SNPs. This notion was reinforced by the substantial, highly significant deficit of nonsense mutations in both EST collections compared to the neutral expectation (Table 1). Remarkably, however, the excess of synonymous over non-synonymous substitutions was less pronounced in cancer ESTs than in normal ESTs. In other words, cancer ESTs showed a significantly greater non-synonymous/synonymous substitution ratio than the normal ESTs (Table 1). (P = 3.7 × 10^-32 ^by Fisher's two-tail exact test) This observation suggests that, compared to the somatic evolution of normal cells, somatic evolution of cancers is characterized by relaxed purifying selection and/or substantial positive selection in some genes. The latter possibility was of special interest because positive selection of somatic mutations in cancers might imply that the change in function of the respective genes was relevant for tumorigenesis and could lead to prediction of previously undetected oncogenes. Therefore we systematically screened cancer and normal EST sequences for indications of positive selection by counting synonymous and non-synonymous substitutions after controlling for sequence quality and subtracting the known SNPs (see Materials and Methods for details).

This screening identified 112 genes with a significant excess of non-synonymous over synonymous substitutions compared to the random expectation in tumors and 37 such genes among normal ESTs (Tables 2 and 3; see Additional file 1 ). The difference between the fractions of genes with a significant excess of non-synonymous substitutions in cancer and normal ESTs was highly statistically significant (P < 10^-7 ^by the Fisher's exact test). Furthermore, a comparison of the mutation spectra in cancer-specific ESTs in the 112 concatenated genes with the mutation spectra of the same genes in ESTs from normal tissues revealed highly significant differences (P < 10^-5^)[36]. Many of the mutated ESTs contain so-called mutational hotspots which, for the purpose of this study, were operationally defined as sites with three or more mutations (Tables 2 and 3; see Additional file 1 ). Specifically, the 112 genes with excess of non-synonymous substitutions in cancer ESTs contained 341 hotspots, whereas the same genes contained 206 hotspots in ESTs from normal tissues. Only 63 hotspots were represented in both sets of ESTs, indicating the presence of a large number of cancer-specific hotspots.

Excess of non-synonymous over synonymous substitutions is considered to be a signature of positive selection [27,35]. For 51 of the 112 genes with such a signature in cancer ESTs, there was also a statistically significant excess of non-synonymous substitutions in a direct comparison with the normal ESTs derived from the same genes (Table 2; see Additional file 1 and Methods for details). Notably, these genes did not seem to have an excess of nonsense mutations (Table 2; see Additional file 1) which suggests that they are not subject to strong selection for loss of function and that at least some of the positively selected amino acid replacements might be associated with gain-of-function.

The methodology employed here dictates that the list of genes with cancer-specific positive selection (CASPS) is dominated by genes that are highly expressed, particularly, in tumors; typically, statistical significance of the positive selection signature could be demonstrated only for genes for which numerous ESTs were available (Table 2; see Additional file 1). Remarkably, however, the list included 30 moderately or even weakly expressed genes that had no synonymous substitutions but had from 4 to 22 non-synonymous substitutions (Table 2; see Additional file 1).

### Cancer connections of CASPS genes and biological implications

Objectively assessing the relevance of a particular gene to tumorigenesis is no easy task. Numerous genes are linked to one or another aspect of cell proliferation, and the expression of many others is perturbed in tumors, which does not necessarily point to an actual role in tumorigenesis. With all these caveats in mind, we nevertheless collated the available data on biological properties of the genes with an apparent signature of positive selection in cancer-derived and normal ESTs and examined their established and potential connections to tumorigenesis. This examination indicated that 42 of the 112 CASPS genes had definitive, experimentally supported connections to tumorigenesis (labeled 'yes' in Table 2; see Additional file 1 ), and for 21 more genes, indirect but strongly suggestive evidence of cancer connections was available (labeled 'likely'). The CASPS genes include those for several proteins involved in DNA repair, programmed cell death, and various forms of signal transduction, among which ubiquitin signaling was particularly prominently represented. All these proteins are directly linked to the control of cell proliferation. Many other CASPS genes do not have such well-defined roles but are substantially overexpressed or amplified in certain types of tumors, which is compatible with involvement in tumorigenesis (Table 2; see Additional file 1).

**Figure 1:**
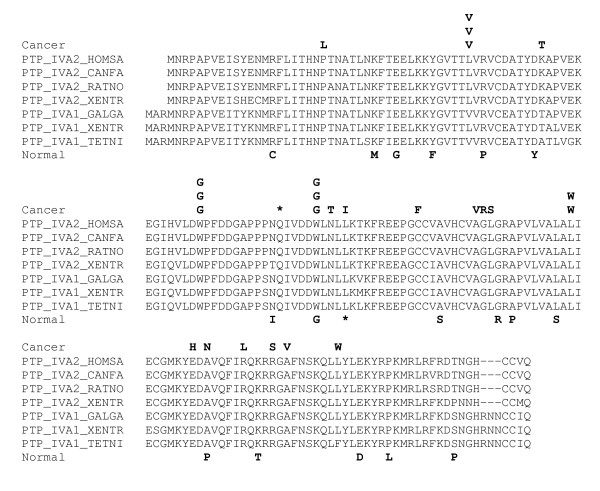
Distribution of non-synonymous substitutions in cancer and normal ESTs. from the protein tyrosine phosphatase IV A2 gene. The substitutions are shown in the form of amino acid replacements. Identical letters in the same position correspond to substitutions from different EST libraries. The alignments were constructed using the MACAW program [44]. Species name abbreviations: CANFA, *Canis familiaris*, GALGA, *Gallus gallus*, HOMSA, *Homo sapiens*, RATNO, *Rattus norvegicus*, XENTR, *Xenopus tropicalis. *TETNI, *Tetraodon nigrovirides*.

**Figure 2:**
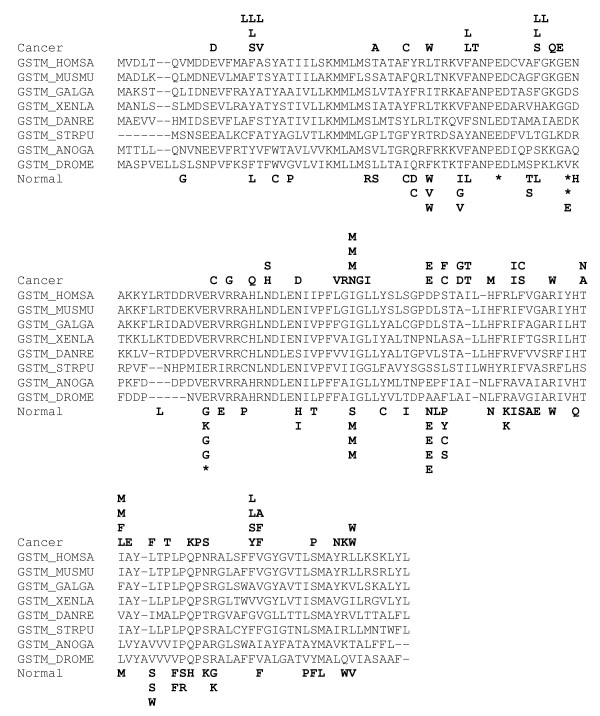
Distribution of non-synonymous substitutions in cancer and normal ESTs. from the microsomal glutathione S transferase gene. The designations are as in Fig. 1. Additional species name abbreviations: ANOGA, *Anopheles gambiae*, DANRE, *Danio rerio*, DROME, *Drosophila melanogaster*, MUSMU, *Mus musculus*, STRPU, *Strongylocentrotus purpuratus*, XENLA, *Xenopus laevis*.

**Figure 3:**
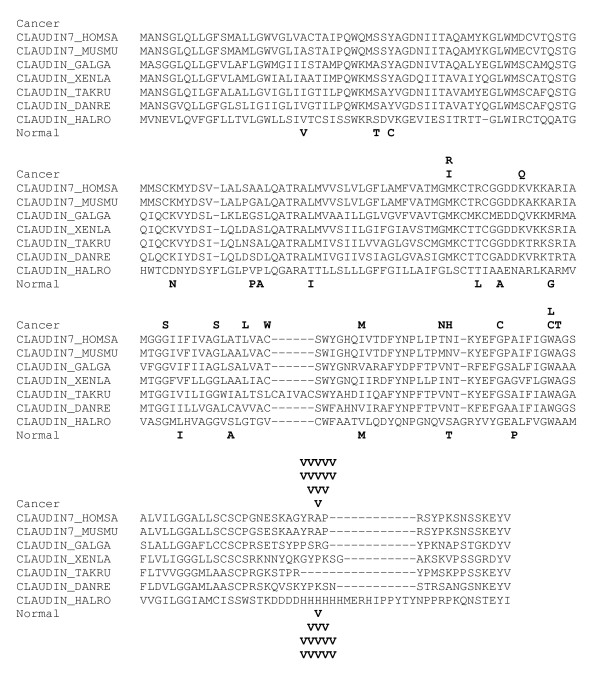
Distribution of non-synonymous substitutions in cancer and normal ESTs. from the claudin 7 gene. The designations are as in Fig. 1. The site with multiple valine substitutions corresponds to a SNP. Additional species name abbreviations: HALRO, *Halocynthia roretzi*, TAKRU, *Takifugu rubripes*.

Figures 1, 2, 3 show the distributions of putative somatic mutations in the sequences of three CASPS genes. These distributions illustrate the complexity of mutational patterns, with distinct spectra seen in cancer and normal ESTs, and a variety of cancer-specific and normal-specific hotspots (Figs. 1,2). Note, however, the presence of a SNP in the same position in both cancer and normal ESTs (Fig. 3)

The CASPS genes included only one well-characterized oncogene, the *ret *protooncogene (Table 2; see Additional file 1). We compared the list of CASPS genes to the comprehensive list of cancer-related set reported in a recent census [13]. In our analysis of EST sequences, mutation spectra with more than 5 mutations were detected for 83 of the 249 cancer-related genes, and 5 of these belonged to the CASPS list. The probability of observing 5 or more genes from a list of 249 among the 112 CASPS genes is ~0.025, suggesting a weak but non-random connection between the CASPS genes detected here and previously characterized cancer-related genes.

## Discussion

The interpretation of the findings on CASPS genes described here requires extreme caution. Although filters were applied to separate somatic mutations from sequencing errors and SNPs (see Methods for details), it is impossible to guarantee that the final list is free of these irrelevant sources of variation. Furthermore, taking into consideration the number of analyzed ESTs, identification of 112 genes with apparent signs of positive selection is, in itself, not particularly surprising. The strongest indication we obtained that some of the CASPS genes are likely to be associated with tumorigenesis is the significant excess of genes with the positive selection signature among cancer ESTs compared to the ESTs from normal tissues (112 against 37). Based on this ratio and assuming that the apparent signature of positive selection in normal ESTs represents the background noise, it should be expected that ~70% of the CASPS genes are, indeed, subject to positive selection during the somatic evolution of tumors. Additionally, the evidence seems convincing for those genes that, individually, showed a significant difference in the non-synonymous to synonymous substitution ratio between cancer and normal ESTs (Table 2; see Additional file 1). From a different perspective, however, it is not certain that somatic mutations in normal tissues are not selected for. Furthermore, it cannot be ruled out that some of the genes that seem to evolve under positive selection in normal tissues are associated with the development of precancerous conditions.

Assuming that there is, indeed, a signal of tumor-specific positive selection in our list of CASPS genes, these are likely to be the tip of the proverbial iceberg of genes that evolve under this regimen in various cancers. Although the current EST database is large and represents most of human genes, it is far from being satisfactory for the purpose of analysis of somatic evolution. In the present study, we had no choice but to lump together ESTs from all types of cancers because the amount of variation in individual tumor types was insufficient for statistical analysis. Furthermore, as already indicated, this analysis is capable of detecting selection only for relatively highly expressed genes. Many genes on our CASPS list and more genes that did not make it contained only several non-synonymous substitutions with no synonymous substitutions. Obviously, the statistical power of the present analysis was insufficient to identify positive selection in these genes.

It is expected that, once the EST or complete cDNA data becomes sufficient for separate analysis of tumors of different origins or, ideally, different cell types and tumor progression stages from individual patients, approaches similar to those employed in this work will provide a wealth of information on somatic evolution of the cancer genome. Establishing ancestor-descendant relationships within individuals will allow one to arrive to definitive conclusions regarding the selection forces in action during tumorigenesis.

## Conclusion

With all due caution, those genes in the CASPS list that met both criteria (significant excess of synonymous mutations and significant difference between cancer and normal ESTs) could be interesting candidates for a detailed analysis aimed at characterization of new oncogenes or genes with other, still poorly understood roles in tumorigenesis. Furthermore, the results of this work emphasize the value of massive EST and mRNA sequencing from various tumor types (or, ideally, from individual tumors) for identifying the complete catalog of genes with a causal role in tumorigenesis.

## Methods

### Data and sequence comparisons

The 25801 non-redundant (all identical sequences were removed) coding sequences (CDS) of human genes from the human genome draft build 35, the April 2004 freeze, obtained at the NCBI ftp server [37] were used as reference sequences to be compared with the EST sequences. The EST sequences were from the dbEST release of August, 2004 [38]. EST Library information was extracted and loaded into a mysql database [39]. Each library was manually curated and assigned to either cancer – related (1413 entries) or normal tissue (1370 entries) bins.

The CDS set was searched against dbEST using the BLASTN program with the default parameters[40]. Unigene Build #173 [41] was used to assign ESTs to a particular locus. Overall, 1844057 Unigene EST hits were identified (Table 1).

The database of single-nucleotide polymorphisms (dbSNP) in the fasta format was downloaded from [42] as of May, 2004 (build 121). Altogether, 20573 non-redundant SNPs were identified in the analyzed set of the CDS to the dbSNP consortium specifications [43] by performing a MEGABLAST search of CDS set against dbSNP (command line: megablast -U T -F m -J F -X 180 -r 10 -q -20 -P 1000 -R T -W 28). All alignments containing gaps were discarded. Using a custom PERL script, the MEGABLAST report was parsed for reliable SNPs by ensuring the identity of the RefSeq sequence with the sequence in dbSNP.

### Identification of probable somatic mutations in EST sequences

To ensure that all analyzed substitutions came from high-quality sequence, a single-nucleotide substitution in an EST sequence was considered a probable somatic mutation if it was flanked, from each side, with 15 nucleotide stretches of perfect identity between the EST sequence and the reference sequence (CDS) and, in addition, a 50-nucleotide stretch with at most 3 mismatches flanking the identical 15 mers on each side. Substitutions that coincided with SNPs from dbSNP and redundant substitutions from the same EST library were discarded. The latter, highly conservative filter was applied to eliminate possible additional, relatively rare SNPs, which are not reported in the current release of dbSNP, and to ensure clonality of all analyzed mutations. The effects of these filters on the analyzed mutations spectra are shown in Table 4. Statistical significance of differences between substitution spectra was determined using a modified χ2 test [36].

### Statistical significance of the differences between ratios of non-synonymous to synonymous substitutions

For each EST, the numbers of synonymous and non-synonymous and substitutions was calculated. These numbers was compared to: i) expected numbers and ii) the numbers for the respective genes in the ESTs from normal (cancer) tissues. The expected values were calculated using a Monte-Carlo random permutation procedure, which was repeated 1000 times for each mutation spectrum. Each step involved random shuffling of transitions/transversions along the appropriate nucleotide sites in the CDS, e.g., for a mutation A->G, the acceptable sites for permutations were those that contained A. Binomial 1-tailed test was used for assessing the statistical significance of non-synonymous vs synonymous substitutions bias in the form:

P(Nobs,n)=∑k=Nobsnn!k!(n−k)!pkqn−k,
 MathType@MTEF@5@5@+=feaafiart1ev1aaatCvAUfKttLearuWrP9MDH5MBPbIqV92AaeXatLxBI9gBaebbnrfifHhDYfgasaacH8akY=wiFfYdH8Gipec8Eeeu0xXdbba9frFj0=OqFfea0dXdd9vqai=hGuQ8kuc9pgc9s8qqaq=dirpe0xb9q8qiLsFr0=vr0=vr0dc8meaabaqaciaacaGaaeqabaqabeGadaaakeaacqWGqbaucqGGOaakcqWGobGtdaWgaaWcbaGaem4Ba8MaemOyaiMaem4CamhabeaakiabcYcaSiabd6gaUjabcMcaPiabg2da9maaqahabaWaaSaaaeaacqWGUbGBcqGGHaqiaeaacqWGRbWAcqGGHaqicqGGOaakcqWGUbGBcqGHsislcqWGRbWAcqGGPaqkcqGGHaqiaaaaleaacqWGRbWAcqGH9aqpcqWGobGtcqWGVbWBcqWGIbGycqWGZbWCaeaacqWGUbGBa0GaeyyeIuoakiabdchaWnaaCaaaleqabaGaem4AaSgaaOGaemyCae3aaWbaaSqabeaacqWGUbGBcqGHsislcqWGRbWAaaGccqGGSaalaaa@5775@

Where *n = N*_*obs*_*+S*_*obs *_is the total number of non-synonymous and synonymous substitutions observed,

p=Nexp⁡n
 MathType@MTEF@5@5@+=feaafiart1ev1aaatCvAUfKttLearuWrP9MDH5MBPbIqV92AaeXatLxBI9gBaebbnrfifHhDYfgasaacH8akY=wiFfYdH8Gipec8Eeeu0xXdbba9frFj0=OqFfea0dXdd9vqai=hGuQ8kuc9pgc9s8qqaq=dirpe0xb9q8qiLsFr0=vr0=vr0dc8meaabaqaciaacaGaaeqabaqabeGadaaakeaacqWGWbaCcqGH9aqpdaWcaaqaaiabd6eaonaaBaaaleaacyGGLbqzcqGG4baEcqGGWbaCaeqaaaGcbaGaemOBa4gaaaaa@361F@ is the ratio of the number of non-synonymous substitutions derived from the Monte-Carlo procedure to the total number of substitutions, and *q = 1-p.*

One-tailed Fisher's exact test was used to assess the significance of the difference between the cancer and normal substitution spectra.

## Abbreviations

CASPS, Cancer-associate positive selection; EST, Expressed sequence tag; SNP, single-nucleotide polymorphism

## Competing interests

The author(s) declare that they have no competing interests.

## Authors' contributions

The study was conceived and designed by EVK and IBR; VNB, MKB, FAK, and IBR performed the computational analysis of the EST mutation data; EVK analyzed the biological aspects of the candidate positively selected genes; VNB wrote the initial draft of the Methods and Results; EVK wrote the final manuscript which was read and approved by all authors.

## Pre-publication history

The pre-publication history for this paper can be accessed here:



## Supplementary Material

Additional File 1 This files contains Tables 2 and 3 together with the corresponding references. Click here for file

## Figures and Tables

**Table 1:** Putative somatic mutations in cancer and normal ESTs.

EST source	Total number of substitutions (N)	Number of synonymous substitutions (Ns)	Number of non-synonymous substitutions (Na)	Number of nonsense substitutions (Nns)	Ns/Na	Nns/N	Ns exp	Na exp	Nns exp	Ns/Na exp	Nns/N exp	p_value χ2 Ns/Na observed vs expected	p_value χ2 Nns/N observed vs expected
Normal	429070	131244	286416	11410	0.46	0.027	111827	301723	15521	0.37	0.036	<10^-10^	<10^-10^
Cancer	253254	74090	172551	6613	0.43	0.026	64719	179818	8717	0.36	0.034	<10^-10^	<10^-10^

**Table 4:** The effects of filtering for SNPs and redundant substitutions on the substitution spectra

	Raw data		After removing SNPs		After removing redundant mutations	
	# spectra (ESTs with substitutions)	# substitutions	# spectra (ESTs with substitutions)	# substitutions	# spectra (ESTs with substitutions)	# substitutions

Cancer	13961	388381	13961	311756	10305	253634
Normal	17116	736031	17116	527018	10352	410800
