# Supplementary material for: Signs of positive selection of somatic mutations in human cancers detected by EST sequence analysis
Source: BMC Cancer. 2006 Feb 9;6:36. doi: 10.1186/1471-2407-6-36 (PMC1431556; doi:10.1186/1471-2407-6-36)
Supplement: Additional File 1 — This files contains Tables 2 and 3 together with the corresponding references. [file 1471-2407-6-36-s1.doc]

Table 2: **Genes with the signature of positive selection detected by analysis of cancer EST libraries^a^**

| Protein | Accession number | Ns observed | Na observed | Nns observed | Ns expected | Na expected | Nns expected | P | P_2x2_ | Cancer  link | Evidence/  comments | Ref |
| --- | --- | --- | --- | --- | --- | --- | --- | --- | --- | --- | --- | --- |
| nuclear pore complex interacting protein | NP_008916 | 2 | 31 | 0 | 9 | 22.4 | 2 | 0.0015 | **0.0011** | Possible | Positive selection in human-chimp divergence | [1];  OMIM 606406 |
| Rad50-interacting protein 1 | NP_068749.2 | 3 | 34 | 1 | 8 | 28 | 2 | 0.023 | **0.0012** | Likely | G(2)/M checkpoint control | [2] |
| ring finger protein 149 | NP_775918.1 | 0 | 23 | 1 | 6 | 18 | 0 | 0.0027 | **0.002** | Likely | The protein has not been experimentally characterized; however, predicted Ub-ligase activity is compatible with cancer involvement. |  |
| glypican 2 | NP_689955.1 | 0 | 11 | 0 | 3 | 8 | 0 | 0.037 | **0.0021** | Yes | Extracellular proteoglycan; implicated in tumor progression and metastasis | [3]  [4]  [5] |
| Microsomal glutathione S-transferase 1 | NP_665707.1 | 9 | 72 | 0 | 19 | 59 | 3 | 0.0019 | **0.0028** | Yes | GSTs play a role in detoxification and cancer drug resistance. Polymorphisms in microsomal GST are associated with laryngeal cancer. | [6] |
| Ubiquinol-cytochrome c reductase binding protein | NP_006285.1 | 3 | 45 | 4 | 9 | 40 | 3 | 0.017 | **0.0028** | Possible | ubiquinol-cytochrome c reductase gene is amplified in high grade breast cancers;  ubiquinol-cytochrome C reductase hinge gene is inactivated in various cancers | [7]  [8] |
| mitochondrial glutamate carrier 1 | NP_078974.1 | 2 | 20 | 1 | 7 | 16 | 1 | 0.019 | **0.0035** | No |  |  |
| origin recognition complex, subunit 1 | NP_004144.2 | 0 | 9 | 0 | 3 | 6 | 0 | 0.034 | **0.0036** | Likely | Regulates DNA replication in a cell-cycle-dependent manner. Concentration of ORC1 is thought to regulate ploidy. | [9] |
| Retinoic acid receptor, gamma | NP_000957 | 0 | 12 | 1 | 3 | 9 | 0 | 0.022 | **0.0044** | Yes | Retinoid signaling is central to tumor progression. Involved in apoptosis regulation. | [10, 11]  [12]  [13] |
| eukaryotic translation initiation factor 4A, isoform 2 | NP_001958.1 | 2 | 21 | 0 | 6 | 17 | 1 | 0.045 | **0.0044** | Likely | Inhibition of the activity of EIF4A by the tumor suppressor Pdcd4 is linked to the transformation-suppressing activity of the latter. | [14]  [15] |
| 3-oxoacid CoA transferase 2 (SCOT) | NP_071403.1 | 0 | 16 | 0 | 3 | 13 | 0 | 0.034 | **0.0058** | Possible | Deficiency in gastric cancers | [16] |
| TBP-associated factor 6 isoform gamma (TAF6) | NP_620834.1 | 9 | 51 | 1 | 19 | 41 | 2 | 0.003 | **0.006** | Likely | Seems to link TFIID to apoptotic signaling | [17]  OMIM 602955 |
| hypothetical protein LOC144363 | XP_370685.1 | 0 | 16 | 0 | 3 | 12 | 1 | 0.024 | **0.0063** | Possible | Predicted NADH-ubiquinone oxidoreductase compelx 1 subunit (LYR family). No specific information on this subunit but complex 1 has a role in oxidative stress response and apoptosis. |  |
| tubulin, beta polypeptide paralog | NP_821080.1 | 3 | 30 | 2 | 10 | 24 | 1 | 0.0037 | **0.0065** | Yes | Major role of microtubules in cancers. Many reports on mutations in β-tubulin being cause of anticancer drug-resistance. However, role of these mutations is considered controversial because in many cases the reported sequences were actually from pseudogenes. | [18] |
| transglutaminase 2 isoform a | NP_004604.2 | 0 | 17 | 1 | 4 | 14 | 1 | 0.015 | **0.0067** | Yes | Activation of transglutaminases by EGF inhibits apoptosis in breast cancer cells. Involved in cell migration and adhesion. | [19]  [20] |
| IGFBP-2-Binding Protein, IIp45 (invasion inhibitory protein) | NP_068752.1 | 13 | 81 | 1 | 24 | 68 | 3 | 0.0037 | **0.0074** | Yes | Inhibits invasion of glioblastoma multiforme cells by binding to insulin-like growth factor which is implicated in invasion. | [21];  OMIM608772 |
| hypothetical protein MGC3123 | NP_803190.1 | 0 | 10 | 0 | 3 | 7 | 0 | 0.026 | **0.008** | Likely | Uncharacterized membrane protein containing the ubiquitin domain |  |
| deoxyguanosine kinase isoform a precursor | NP_550438.1 | 0 | 15 | 1 | 3 | 12 | 1 | 0.026 | **0.008** | Likely | Mitochondrial protein, mutations result in depletion of mitochondrial DNA; low expression in cancer cell line resistant to 9-β-D-arabinofuranosylguanine and other nucleotide analogs. | [22]  [23] |
| ribosomal protein L35a | NP_000987.2 | 11 | 64 | 2 | 21 | 54 | 3 | 0.0058 | **0.0085** | Yes | Over-expression inhibits apoptosis, over-expressed in glioblastoma multiforme. | [24, 25] |
| hypoxia-inducible factor 1, α subunit isoform 1 | NP_001521.1 | 9 | 51 | 4 | 16 | 46 | 2 | 0.041 | **0.0092** | Yes | Activator of transcription of hypoxia-induced genes and tumor angiogenesis. Considered a major anti-cancer drug target. | [26]  [27] |
| chromosome 19 open reading frame 10 | NP_061980.1 | 8 | 52 | 3 | 14 | 47 | 2 | 0.042 | **0.0094** | Unknown | Uncharacterized protein conserved in vertebrates |  |
| cullin 1 | NP_003583.2 | 6 | 63 | 2 | 17 | 52 | 2 | 0.00097 | **0.0099** | Yes | Subunit of ubiquitin-ligase complexes; involved in cell-cycle regulation, G1-to-S phase progression is accelerated in CUL1 mutants. Considered a target for anticancer drugs. Role in proper chromosomal segregation, inhibition causes aneuploidy and transformation. | [28]  [29]  [30];  OMIM 603134 |
| transforming growth factor beta 1 induced transcript 4 isoform 2 (TSC-22) | NP_006013.1 | 0 | 12 | 0 | 3 | 9 | 0 | 0.043 | **0.01** | Yes | A component of the proliferator-activated receptor γ and transforming growth factor β involved in epithelial cell growth and differentiation. Down-regulation of TSC-22 considered important for development of salivary gland cancer; candidate drug target. Putative tumor suppressor. | [31-33] |
| TBP-associated factor 6 isoform α | NP_005632.1 | 13 | 52 | 1 | 20 | 45 | 2 | 0.038 | **0.011** | Yes | Part of a signaling pathway connecting apoptosis with transcription regulation | [17] |
| sulfatase modifying factor 2 | NP_056226.1 | 14 | 79 | 1 | 24 | 66 | 4 | 0.0052 | **0.012** | Possible | C(α)-formylglycine generating enzyme responsible for post-translational modification of the active site of multiple sulfatases. Steroid sulfatase implicated in tumor development. | [34] [35] |
| brevican isoform 2 | NP_940819.1 | 20 | 99 | 5 | 32 | 88 | 5 | 0.0082 | **0.012** | Yes | Chondroitin sulfate proteoglycan up-regulated in gliomas. Seems to have role in glioma invasion. | [36]  [37, 38] |
| claudin 7 | uspe | 2 | 27 | 0 | 7 | 21 | 1 | 0.016 | **0.012** | Yes | Tight junction membrane protein implicated in spread of breast and prostate cancers. | [39]  [40] |
| hypothetical protein FLJ36031 | NP_787080.1 | 2 | 23 | 1 | 7 | 19 | 0 | 0.023 | **0.012** | Unknown |  |  |
| mitogen-activated protein kinase 7 isoform 1 | NP_620602.1 | 0 | 9 | 0 | 3 | 6 | 0 | 0.031 | **0.012** | Yes | Regulator of cell proliferation | [41, 42] |
| mitochondrial ribosomal protein S21 | NP_114107.1 | 5 | 32 | 1 | 10 | 26 | 1 | 0.028 | **0.015** | No |  |  |
| hypothetical protein HSPC268 | NP_932068.1 | 0 | 13 | 0 | 3 | 10 | 0 | 0.027 | **0.017** | Unknown | Small all-helical protein conserved in animals, plants, and fungi. Homolog of yeast Fmc1p which appears to be involved in the assembly/stabilization of mitochondrial ATPase under stress conditions. | [43] |
| protein tyrosine phosphatase type IVA, member 2 isoform 1 | NP_003470.1 | 3 | 25 | 1 | 8 | 20 | 2 | 0.03 | **0.017** | Yes | Oncogene, stimulates cell proliferation and invasiveness; candidate target for anticancer drugs | [44] [45]  [46] |
| NADH dehydrogenase (ubiquinone) 1 beta subcomplex 2, 8kDa precursor | NP_004537.1 | 1 | 19 | 0 | 4 | 15 | 1 | 0.043 | **0.019** | Possible | No direct data but complex I plays a role in oxidative stress response and apoptosis |  |
| caspase 4 isoform alpha precursor | NP_001216.1 | 4 | 34 | 1 | 9 | 28 | 2 | 0.025 | **0.02** | Yes | Key role in apoptosis; this particular form is implicated in cancer progression | [47] [48] |
| chronic myelogenous leukemia tumor antigen 66 | NP_116258.1 | 1 | 22 | 0 | 6 | 16 | 0 | 0.0047 | **0.021** | Yes | A broadly immunogenic tumor antigen highly expressed in solid tumors and hematopoietic malignancies. Proposed target for antigen-specific immunotherapy. | [49]  OMIM 606109 |
| regulatory subunit PR 53 of protein phosphatase 2A isoform d | NP_821070.1 | 1 | 19 | 0 | 6 | 14 | 1 | 0.012 | **0.022** | Yes | Phosphatase 2A is a major negative regulator of cell cycle progression | [50] [51] |
| apolipoprotein E precursor | NP_000032.1 | 10 | 43 | 0 | 16 | 34 | 3 | 0.027 | **0.023** | Yes | Implicated in invasiveness of prostate cancer; cell proliferation and survival in ovarian cancer | [52]  [53] |
| NADH dehydrogenase (ubiquinone) 1 alpha subcomplex4, 9kDa | NP_002480.1 | 7 | 47 | 2 | 13 | 41 | 2 | 0.037 | **0.026** | Possible | No direct data but complex I plays a role in oxidative stress response and apoptosis |  |
| TCF3 (E2A) fusion partner (in childhood Leukemia) | NP_037474.1 | 10 | 45 | 1 | 16 | 38 | 2 | 0.039 | **0.026** | Yes | Common fusion in childhood leukemia, suggesting involvement in leukemogenesis; implicated in neuronal cell death. | [54] [55] |
| tumor necrosis factor receptor superfamily, member 10b isoform 1 precursor | NP_003833.3 | 0 | 17 | 1 | 5 | 13 | 1 | 0.0047 | **0.028** | Yes | Major role in apoptosis regulation; mutations and translocations in head and neck carcinomas and non-small cell lung cancers. | [56] [57]; [56] |
| peroxiredoxin 2 isoform a | NP_005800.3 | 8 | 45 | 3 | 15 | 39 | 2 | 0.026 | **0.028** | Yes | Central role in oxidative stress response, p53-dependent, changed expression in cancers | [58] [59] |
| mitochondrial ribosomal protein L27 isoform a | NP_057588.1 | 0 | 8 | 0 | 3 | 6 | 0 | 0.034 | **0.028** | No |  |  |
| complement component 1 inhibitor precursor | NP_000053.1 | 4 | 24 | 0 | 8 | 19 | 1 | 0.046 | **0.028** | No |  |  |
| phospholipase D3 | NP_036400.1 | 3 | 25 | 2 | 10 | 20 | 1 | 0.0073 | **0.032** | Likely | This particular gene not studied in detail but phospholipase D is a critical regulator of cell proliferation and tumor progression whose expression is changed in many tumors. | [60] |
| serine carboxypeptidase, vitellogenic-like (CPVL) | NP_061902.1 | 3 | 30 | 2 | 11 | 24 | 2 | 0.0042 | **0.033** | Unknown | Highly expressed in macrophages, could be involved in inflammation protease cascade but not studied in any detail. | [61] |
| nuclear receptor subfamily 1, group H, member 3 | NP_005684.1 | 12 | 60 | 0 | 19 | 51 | 2 | 0.023 | **0.033** | Yes | Lipid metabolism regulator; also regulation of cell proliferation, antiproliferative effect in prostate cancer cells. Retinoid signaling is central to tumor progression | [62]  [63] [64] |
| electron transfer flavoprotein, alpha polypeptide | NP_000117.1 | 19 | 97 | 1 | 29 | 85 | 3 | 0.014 | **0.039** | Possible | Involved in regulation of ROS production although no specific cancer links. | [65] |
| ubiquitin protein ligase E3B isoform α | NP_904324.1 | 1 | 19 | 0 | 5 | 14 | 1 | 0.024 | **0.04** | Likely | Ubiquitin signaling plays a central role in cell proliferation control although there is no data on this particular Ub ligase | [66] |
| glutathione transferase zeta 1 isoform 1 | NP_665877.1 | 8 | 53 | 3 | 21 | 42 | 1 | 0.00031 | **0.041** | Possible | GSTs play a role in detoxification and cancer drug resistance | [67]  OMIM 603758 |
| protein phosphatase 1, regulatory (inhibitor) subunit 12A | NP_002471.1 | 0 | 8 | 0 | 2 | 5 | 1 | 0.05 | **0.042** | Yes | Regulatory subunit of myosin phosphatase. Implicated in cell invasion. Apparently phosphorylated by ROK and thus involved in angiogenesis and tumor growth. | [68] [69] |
| karyopherin beta 1 | NP_002256.2 | 0 | 9 | 0 | 3 | 6 | 0 | 0.048 | 0.052 | Possible | No direct data but nuclear transport is regulated in a cell-cycle dependent manner; involvement in cell cycle control likely. | [70] |
| NADH dehydrogenase (ubiquinone) 1 alpha subcomplex, 5 | NP_004991.1 | 5 | 39 | 3 | 13 | 32 | 1 | 0.0044 | 0.056 | Possible | No direct data but complex I plays a role in oxidative stress response and apoptosis | [65] |
| protein kinase Myt1 isoform 1 | NP_004194.3 | 4 | 32 | 0 | 9 | 26 | 1 | 0.034 | 0.059 | Yes | Cell cycle regulator, phosphorylates and inactivates CDC2, inhibits G2/M transition | [71] [72] |
| 3D3/lyric/  Metadherin | NP_848927.1 | 9 | 56 | 4 | 16 | 50 | 4 | 0.035 | 0.06 | Yes | Over-expression is associated with metastatic breast cancer; surface protein that appears to directly mediate lung metastasis of breast cancer | [73] |
| hypothetical protein FLJ10204 | NP_060494.1 | 4 | 27 | 1 | 8 | 22 | 1 | 0.047 | 0.06 | Unknown | Uncharacterized protein conserved in most eukaryotes |  |
| S100 calcium-binding protein A10 | NP_002957.1 | 4 | 27 | 1 | 9 | 23 | 1 | 0.048 | 0.06 | Yes | Involved in the regulation of cell cycle progression and differentiation. Typical markers in many cancers. | [74, 75] [76] |
| APEX nuclease | NP_542379.1 | 6 | 40 | 6 | 12 | 37 | 3 | 0.041 | 0.064 | Yes | A major apurinic/apyrimidinic endonuclease in base excision and nucleotide excision repair; involved in DNA fragmentation during apoptosis. SNPs are associated with increased cancer risk. Elevated activity in some cancers. | [77] [78]  [79] |
| cyclin G2 | NP_004345.1 | 1 | 15 | 1 | 6 | 11 | 1 | 0.01 | 0.067 | Yes | Critical for cell cycle regulation, damage-induced | [80] [81] |
| DEAD (Asp-Glu-Ala-Asp) box polypeptide 27 | NP_060365.6 | 0 | 9 | 0 | 3 | 6 | 0 | 0.049 | 0.068 | Possible | No specific data but regulatory function likely. |  |
| COBL-like 1 | NP_055715.2 | 0 | 10 | 0 | 3 | 7 | 0 | 0.046 | 0.071 | Unknown |  |  |
| hypothetical protein BC004923 | NP_149098.1 | 4 | 32 | 1 | 10 | 26 | 1 | 0.016 | 0.073 | Unknown | Putative translation factor of the Obg family of RNA-binding GTPases |  |
| exonuclease 1 isoform a | NP_003677.3 | 0 | 20 | 1 | 7 | 13 | 1 | 0.00032 | 0.075 | Likely | Function in repair and recombination, interacts with WRN. Mutations found in hereditary nonpolyposis colon cancer but cancer link disputed because some of the same variants found in normal population | [82] [83] [84];  OMIM 606063 |
| serine/arginine repetitive matrix 1 (Srm160) | **NP_005830.2** | 16 | 71 | 3 | 24 | 63 | 3 | 0.031 | 0.077 | Possible | Splicing regulator, associates with the DEK protein, which is involved in translocations in acute myeloid leukemias, and with proto-oncogene TLS-FUS | [85] [86] |
| WD SOCS-box protein 1 isoform 1 | NP_056441.6 | 0 | 10 | 0 | 3 | 7 | 1 | 0.038 | 0.077 | Yes | Regulator of cytokine signaling and ubiquitin-dependent protein degradation; some of the SOCS genes are hypermethylated and silenced in ovarian and breast cancers. | [87] [88] |
| zinc finger protein 24 (KOX 17) | NP_008896.1 | 4 | 30 | 2 | 9 | 25 | 1 | 0.028 | 0.081 | Possible | Probable transcription regulator but no data on this particular protein. |  |
| splicing factor, arginine/serine-rich 5 | NP_008856.1 | 1 | 13 | 0 | 5 | 9 | 0 | 0.031 | 0.084 | Likely | Probable involvement in regulation of alternative splicing important for oncogenesis. | [89] |
| 3' exoribonuclease | NP_699163.2 | 0 | 11 | 1 | 3 | 9 | 1 | 0.048 | 0.084 | Likely | A primary candidate for the exonuclease that initiates rapid decay of histone mRNA upon completion and/or inhibition of DNA replication. Also involved in regulation of post-transcriptional gene silencing. | [90, 91] |
| chromosome 10 open reading frame 61 | NP_056446.1 | 3 | 29 | 0 | 7 | 24 | 1 | 0.036 | 0.087 | Unknown | Uncharacterized protein conserved in animals |  |
| Protein kinase D2 | NP_057541.2 | 2 | 25 | 0 | 8 | 19 | 1 | 0.0066 | 0.088 | Likely | Regulates basolateral membrane protein exit from trans-Golgi network. Negative modulator of the c-Jun N-terminal kinase (JNK) signaling pathway. | [92]  [93] |
| Multisynthetase complex auxiliary component p38 | NP_006294.2 | 14 | 76 | 2 | 22 | 67 | 3 | 0.027 | 0.088 | Unknown | Auxiliary subunit of the multisynthetase aminoacyl-tRNA complex, contains a GST domain. | [94] |
| zinc finger protein 585B | NP_689492.2 | 0 | 11 | 1 | 3 | 8 | 0 | 0.025 | 0.094 | Possible | KRAB-containing C2H2 multiple Zn-finger protein, probable transcriptional regulator |  |
| mitochondrial ribosomal protein L50 | NP_061924.1 | 4 | 24 | 0 | 8 | 19 | 1 | 0.048 | 0.098 | No |  |  |
| N-acylsphingosine amidohydrolase (acid ceramidase) | NP_004306.2 | 9 | 62 | 0 | 19 | 50 | 2 | 0.0019 | 0.1 | Yes | Over-expression protects cells from TNF-induced death. Over-expressed but apparently not mutated in prostate cancer but thought to be important for prostate tumorigenesis. | [95] [96]  OMIM 228000 (Farber lipogranulomatosis) |
| Eukaryotic translation initiation factor 3, subunit 2 beta, 36kDa | NP_003748.1 | 75 | 309 | 10 | 93 | 288 | 13 | 0.013 | 0.1 | Yes | TGFβ receptor-interacting protein 1, phosphorylated by TGFβ receptor. Amplified in advanced prostate cancer. | [97] [98] |
| chromosome 2 open reading frame 4/Memo | NP_057039.1 | 2 | 22 | 0 | 6 | 18 | 1 | 0.049 | 0.1 | Yes | Predicted dioxygenase highly conserved in eukaryotes and archaea. Apparently controls cell migration by relaying extracellular chemotactic signals to the microtubule cytoskeleton. | [99] |
| NADH-ubiquinone oxidoreductase Fe-S protein 7 | NP_077718.2 | 12 | 34 | 1 | 19 | 26 | 2 | 0.017 | 0.11 | Unknown |  |  |
| glutamate-ammonia ligase (glutamine synthase) | NP_002056.2 | 22 | 113 | 2 | 32 | 100 | 5 | 0.016 | 0.12 | Likely | Upregulated in liver tumors, particularly, those with β−catenin mutations. | [100] [101] |
| surfeit 5 isoform b | NP_598395.1 | 0 | 8 | 0 | 3 | 5 | 1 | 0.043 | 0.12 | Possible | Subunit of the SRB-TBP (Mediator) complex involved in regulation of transcription initiation, homolog of yeast SRB6 | [102] [103] |
| fibrinogen-like 1 precursor | NP_004458.3 | 1 | 13 | 0 | 4 | 9 | 1 | 0.044 | 0.12 | Yes | Liver-specific protein, mutated and/or down-regulated in hepatocellular carcinoma, thought to be a tumor suppressor. | [104] [105] |
| major vault protein | NP_059447.2 | 28 | 123 | 9 | 44 | 112 | 4 | 0.0045 | 0.13 | Yes | Typically overexpressed in multidrug-resistant cells, predictor of poor outcome of chemotherapy. Deletion in acute myeloid leukemia. | [106] [107]  OMIM 605088; |
| phosphatidylethanolamine N-methyltransferase isoform 2 | NP_009100.2 | 14 | 59 | 0 | 22 | 49 | 3 | 0.02 | 0.13 | Likely | Low expression in liver tumors; loss of expression seems to be associated with clinical progression. | [108] [109]  [110] |
| WD repeat domain 21 isoform 1 | NP_056419.2 | 6 | 42 | 1 | 11 | 36 | 2 | 0.045 | 0.14 | Possible | General role in signal transduction likely; no specific data |  |
| neuropathy target esterase | NP_006693.2 | 3 | 23 | 1 | 8 | 18 | 1 | 0.018 | 0.15 | Possible | Plays a central role in membrane lipid homeostasis. | [111] |
| ribosomal protein L28 | NP_000982.2 | 52 | 150 | 3 | 65 | 135 | 5 | 0.027 | 0.16 | Possible | Expression decreased in colorectal cancers | [112] |
| SP110 nuclear body protein isoform b;interferon-induced protein 41/75 | NP_004501.2 | 3 | 37 | 1 | 12 | 28 | 1 | 0.00038 | 0.17 | Unknown | Nucleolar protein, possible role in ribosomal biogenesis | [113] |
| stromal cell protein | NP_061333.1 | 1 | 15 | 0 | 4 | 11 | 1 | 0.033 | 0.17 | Unknown | Highly conserved multitransmembrane protein; involved in activation of RAG1 in lymphoid tissue | [114] |
| hypothetical protein FLJ11184 | NP_060822.1 | 1 | 16 | 0 | 4 | 11 | 1 | 0.033 | 0.17 | Unknown | Helical protein conserved in nearly all eukaryotes |  |
| CDP-diacylglycerol--inositol 3-phosphatidyltransferase isoform 1 | NP_006310.1 | 11 | 55 | 5 | 18 | 51 | 3 | 0.046 | 0.20 | Yes | Phosphatidylinositol breakdown products are ubiquitous second messengers that function downstream of many G protein-coupled receptors and tyrosine kinases regulating cell growth. A inhibitor of this enzyme has a cytostatic effect on oral squamous cell carcinoma cells. | [115] [116]  [117] |
| hypothetical protein MGC31967 | NP_777583.1 | 0 | 11 | 1 | 3 | 9 | 0 | 0.028 | 0.21 | Unknown |  |  |
| protein kinase C binding protein 1 isoform b | NP_036540.3 | 1 | 18 | 0 | 5 | 3 | 1 | 0.029 | 0.21 | Yes | Cutaneous T-cell lymphoma-associated antigen. Probable transcriptional regulator (Bromo domain+2 MYND finger domains (Ub ligase?) Also expressed in many other tumors. | [118] [119]  [120] |
| tumor necrosis factor type 1 receptor associated protein | NP_057376.1 | 87 | 334 | 14 | 108 | 313 | 14 | 0.0094 | 0.26 | Yes | Mitochondrial HSP90 chaperone TRAP1. Suppression of TRAP1 expression might play an important role in the induction of ROS-dependent apoptosis. | [121, 122] |
| hypothetical protein MGC2494 | NP_076422.1 | 2 | 14 | 0 | 6 | 10 | 1 | 0.026 | 0.26 | Unknown | Predicted protein methyltransferase |  |
| Close paralog of Elongin B (Elongin 18 kDa subunit) | XP_171447.1 | 0 | 8 | 0 | 3 | 5 | 0 | 0.02 | 0.27 | Yes | Associates with VHL, a classical tumor suppressor, to regulate expression of hypoxia-inducible mRNAs | [123] [124]  [125] |
| POMZP3 fusion protein isoform 1 | NP_036362.2 | 6 | 31 | 0 | 12 | 24 | 1 | 0.017 | 0.28 | Unknown | Gene fusion of the nuclear pore membrane protein POM121 with zona pellucida protein ZP3. |  |
| abl interactor 2 | NP_005750.3 | 2 | 20 | 0 | 6 | 15 | 2 | 0.035 | 0.28 | Likely | Interacts with Abl and phosphorylated by it; involved in cytoskeleton reorganization in response to growth factors; probable regulator of cell motility. | [126] [127] |
| hypothetical protein XP_370868 | XP_370868.1 | 8 | 31 | 0 | 13 | 25 | 1 | 0.037 | 0.32 | Unknown | Uncharacterized protein conserved in vertebrates |  |
| hypothetical protein MGC3234 | NP_076436.2 | 22 | 76 | 2 | 32 | 64 | 4 | 0.011 | 0.33 | unknown | Predicted glycosyl hydrolase, family 18 (possible role in invasion?) |  |
| sideroflexin 3, predicted cation transporter | NP_112233.2 | 3 | 36 | 0 | 10 | 27 | 2 | 0.0025 | 0.34 | Unknown | Implicated in sideroblastic anemia but no demonstrated cancer connections | [128] |
| zinc finger, DHHC domain containing 4 | NP_060576.1 | 6 | 51 | 1 | 13 | 43 | 3 | 0.011 | 0.34 | Unknown |  |  |
| carnitine acetyltransferase isoform 1 precursor | NP_000746.2 | 5 | 37 | 0 | 11 | 29 | 2 | 0.014 | 0.37 | No | Key enzyme of carnitine metabolism, regulates AcCoA/CoA ratio. No clear cancer links. | [129] |
| guanidinoacetate N-methyltransferase isoform b | NP_620279.1 | 3 | 29 | 1 | 9 | 22 | 1 | 0.0058 | 0.4 | Unknown | Last step in creatine biosynthesis. | [130] |
| hypothetical protein MGC10993 | NP_085054.1 | 4 | 41 | 0 | 11 | 33 | 1 | 0.0073 | 0.42 | Unknown | Predicted multitransmembrane protein conserved in animals |  |
| phosphatidic acid phosphatase type 2C isoform 2 | NP_803545.1 | 4 | 34 | 2 | 11 | 28 | 1 | 0.0059 | 0.43 | Possible | Involved in receptor-activated signal transduction mediated by phospholipase and in ceramide metabolism. | [131] |
| gemin4 | NP_056536.1 | 2 | 9 | 0 | 5 | 6 | 1 | 0.049 | 0.5 | Yes | SMN complex subunit, involved in splicing.  Implicated in hepatocellular carcinoma growth control. | [132] [133] |
| ninjurin 1 (nerve-injury-induced protein) | NP_004139.1 | 1 | 35 | 0 | 9 | 26 | 1 | 0.00019 | 1 | Possible | Role in nerve regeneration and, possibly, growth of other tissue; located in the candidate region for the hereditary cancer predisposition syndrome multiple self-healing squamous epithelioma | [134, 135]  OMIM 602062 |
| Sirtuin 6 | NP_057623.1 | 10 | 45 | 1 | 19 | 36 | 2 | 0.0063 | 1 | Likely | This particular gene has not been studied in detail but surtuins in general have a major role in regulation of genome stability. | [136] |
| acidic (leucine-rich) nuclear phosphoprotein 32 family, member B | NP_006392.1 | 29 | 163 | 3 | 43 | 147 | 5 | 0.0075 | 1 | Maybe | Involved in cell proliferation regulation, in particular, in the brain. Highly conserved in most eukaryotes. | [137] |
| ret proto-oncogene isoform a | NP_066124 | 4 | 27 | 1 | 9 | 22 | 1 | 0.027 | 1 | Yes | Receptor tyrosine kinase, mutations implicated in a variety of cancers, particularly, endocrine ones. | [138] |
| candidate tumor suppressor in ovarian cancer 2 | NP_543012.1 | 3 | 17 | 0 | 7 | 12 | 1 | 0.037 | 1 | Yes | Located in a region with frequent allelic loss in breast and ovarian cancers; down-regulated by retinoids; predicted α/β-hydrolase conserved in most eukaryotes; candidate tumor suppressor | [139] |
| claudin 3 | NP_001297.1 | 25 | 73 | 3 | 33 | 63 | 5 | 0.041 | 1 | Yes | Tight junction membrane protein implicated in spread of breast and prostate cancers. | [39]  [40] |
| LOC124402 | NP_660296.1 | 0 | 12 | 0 | 3 | 9 | 0 | 0.043 | NA | Unknown | Uncharacterized protein conserved in coelomates |  |
| DNA directed RNA polymerase III polypeptide K | NP_057394.1 | 0 | 16 | 0 | 3 | 13 | 0 | 0.043 | NA | Possible | Small essential subunit of RNAP III | [140] |

^a^The quantities in the table are as follows: Ns observed, observed number of synonymous substitutions, Na observed, observed number of non-synonymous substitutions, Nns observed, observed number of nonsense mutations, Ns expected, expected number of synonymous substitutions , Na expected, expected number of non-synonymous substitutions, Nns expected, expected number of nonsense substitutions (for the estimation of expected numbers, see Materials and Methods), P, probability that the excess of non-synonymous over synonymous substitutions is due to chance (binomial 1-tail test), P2x2, probability that the difference in the ratios of non-synonymous to synonymous substitutions in the given in the cancer ESTs and normal ESTs is due to chance (Fisher’s exact 2-tail test; bold type denotes significant values, P<0.05).

Table 3: **Genes with a signature of positive selection detected by analysis of EST libraries from normal tissues^a^**

| Protein | Accession number | Ns observed | Na observed | Nns observed | Ns expected | Na expected | Nns expected | P | P_2x2_ | Cancer  link | Evidence/comments | Reference |
| --- | --- | --- | --- | --- | --- | --- | --- | --- | --- | --- | --- | --- |
| similar to RIKEN cDNA 2410004L22 | XP_050564.2 | 0 | 13 | 0 | 4 | 9 | 0 | 0.011 | **0.000059** | Unknown | Uncharacterized protein conserved in vertebrates |  |
| regulator of G-protein signalling 2, 24kDa | NP_002914.1 | 0 | 15 | 0 | 3 | 12 | 1 | 0.027 | **0.00028** | yes | Involved in G-protein-coupled signaling including the G0 to G1 switch in cell cycle. Proposed to promote metastasis. | [141]  [142]  [143] |
| N-acetyl-glucosamine-1-phosphotransferase, γ subunit | NP_115909.1 | 5 | 30 | 0 | 9 | 24 | 1 | 0.048 | **0.00033** | Possible | Expression seems to be elevated in some tumors | [144]  [145] |
| MMS19-like (MET18 homolog, S. cerevisiae) | NP_071757.2 | 2 | 15 | 0 | 6 | 11 | 1 | 0.04 | **0.0027** | Yes | Function in repair; binds nuclear receptor coactivator RAC3 which is amplified in breast cancer. | [146] |
| protein kinase D3 | NP_005804.1 | 0 | 12 | 0 | 3 | 9 | 0 | 0.043 | **0.0043** | Likely | Regulates basolateral membrane protein exit from trans-Golgi network; rapidly activated by protein kinase C in small cell lung cancer lines. | [147]  [92] |
| alpha-1,3-mannosyltransferase ALG2 isoform 2 | NP_932077.1 | 0 | 12 | 0 | 3 | 9 | 0 | 0.046 | **0.005** | No |  |  |
| killer cell lectin-like receptor subfamily B, member 1 | NP_002249.1 | 0 | 11 | 0 | 3 | 7 | 0 | 0.031 | **0.0065** | No |  |  |
| General transcription factor IIE, polypeptide 2, beta 34kDa | NP_002086.1 | 0 | 13 | 1 | 3 | 11 | 0 | 0.042 | **0.01** | Likely | Binds MDM2, participates in inhibition of p53-mediated transcription by MDM2 | [148] |
| acid phosphatase 1 isoform c | NP_004291.1 | 2 | 33 | 0 | 8 | 26 | 1 | 0.0071 | **0.013** | Possible | No specific data but cancer involvement is plausible given the role in signal transduction. |  |
| atlastin; guanylate-binding protein 3 | NP_056999.2 | 1 | 18 | 0 | 4 | 15 | 1 | 0.049 | **0.013** | Possible | Membrane GTPase of the dynamin-Mx family, probably, involved in Golgi membrane dynamics | [149] |
| ribosomal protein P2 | NP_000995.1 | 22 | 106 | 1 | 32 | 93 | 5 | 0.015 | **0.022** | Likely | Accessory ribosomal protein, activated by GPCR-dependent phosphorylation. Antisense oligos against P2 slow growth of cancer cells, apparently through multiple changes in the proteome. | [150]  [151] |
| similar to 60S ribosomal protein L29 (Cell surface heparin binding protein HIP) | XP_063630.5 | 1 | 17 | 0 | 4 | 13 | 0 | 0.039 | **0.036** | Likely | Involved in cell-cell interactions, affects proliferation, down-regulated in colon cancer cells, implicated in anticancer drug-induced apoptosis. | [152]  [153] |
| similar to cytokine | XP_371044.1 | 1 | 25 | 0 | 8 | 17 | 1 | 0.00068 | **0.043** | Likely | No specific data but cytokines have a major role in control of tumor growth. | [154] |
| vinexin β (SH3-containing adaptor molecule-1) | NP_005766.2 | 2 | 20 | 0 | 6 | 16 | 0 | 0.044 | 0.058 | likely | Regulates cytoskeleton organization, involved in EGF/ERK signaling | [155]  [156] |
| nuclear receptor-binding factor 1 | NP_057095.1 | 1 | 16 | 1 | 5 | 13 | 0 | 0.03 | 0.063 | Unknown | homolog of yeast 2-enoyl thioester reductase | [157] |
| Lysosomal acid phosphatase 2 precursor | NP_001601.1 | 3 | 21 | 0 | 7 | 17 | 1 | 0.05 | 0.067 | Likely | No data on this particular protein but acid phosphatases are up-regulated in many tumors including being markers for prostate cancer. | [158] |
| CLN3 protein | NP_000077.1 | 8 | 46 | 2 | 15 | 40 | 1 | 0.027 | 0.14 | Yes | Lysosomal protein mutated in Batten disease. Implicated in neuronal differentiation. Over-expressed in many cancers, inhibition of expression inhibits growth of cancer cells. | [159]  [160] |
| LR8 protein | NP_054739.2 | 9 | 44 | 2 | 15 | 38 | 2 | 0.041 | 0.17 | Possible | Paralog of a purported hepatocellular carcinoma-associated antigen |  |
| ATPase, H+ transporting, lysosomal, V0 subunit c | NP_001685.1 | 3 | 19 | 0 | 7 | 15 | 0 | 0.041 | 0.23 | Likely | No data on this particular subunit but in general proton pumps are up-regulated in tumors to counter-act intracellular acidification. | [161] |
| DNA directed RNA polymerase II polypeptide E | NP_002686.2 | 6 | 35 | 1 | 15 | 23 | 3 | 0.00048 | 0.3 | Possible | Involved in transcription regulation, cell cycle regulation and interaction with activating factors.  However, no specific data. | [162] |
| TAP binding protein-like | NP_060479.2 | 3 | 28 | 1 | 8 | 24 | 1 | 0.041 | 0.32 | unknownw | links MHC class I molecules to the transporter associated with antigen processing (TAP) in the | [163] |
| similar to Fatty acid-binding protein, epidermal (E-FABP) (Psoriasis-associated fatty acid-binding protein homolog) (PA-FABP) | XP_370729.2 | 17 | 75 | 0 | 24 | 63 | 5 | 0.026 | 0.35 | Possible | Significant changes in expression in bladder cancers | [164] |
| dynein 2 light intermediate chain isoform 2 | NP_056337.1 | 1 | 15 | 0 | 4 | 11 | 0 | 0.03 | 0.35 | Likely | No specific data but cancer link plausible given the role of microtubules. |  |
| hypothetical protein XP_371479 | XP_371479.1 | 8 | 55 | 2 | 17 | 45 | 3 | 0.0045 | 0.38 | Possible | NADH:ubiquinone oxidoreductase 17.2 kD subunit; No specific information on this subunit but complex 1 has a role in oxidative stress response and apoptosis. |  |
| M-phase phosphoprotein 10 - U3 small nucleolar ribonucleoprotein component | NP_005782.1 | 1 | 21 | 0 | 4 | 17 | 1 | 0.045 | 0.47 | Possible | No specific data but cancer link is plausible given involvement in splicing regulation and cell-cycle-dependent subcellular localization | [165] |
| gemin4 | NP_056536.1 | 5 | 25 | 0 | 10 | 19 | 1 | 0.035 | 0.5 | Yes | SMN complex subunit, involved in splicing.  Implicated in hepatocellular carcinoma growth control. | [132]  [133] |
| thymosin-like 3 | NP_898870.1 | 0 | 17 | 1 | 5 | 12 | 2 | 0.0033 | 1 | Likely | No specific data but thymosins are involved in control of tumor growth. | [166] |
| Fc fragment of IgG, high affinity Ia, receptor for (CD64) | NP_000557.1 | 2 | 18 | 2 | 7 | 14 | 1 | 0.017 | 1 | Yes | Critical for macrophage activation, target for immunotherapy. Relevant to immune response to cancer not transformation/tumor progression per se. | [167]  [168] |
| Inhibitor of DNA binding 3 | NP_002158.2 | 2 | 25 | 0 | 7 | 20 | 0 | 0.017 | 1 | Likely | Inhibits binding of HLH transcription factors with chromatin by sequestering them in the nucleus. Over-expression stimulates angiogenesis. | [169]  [170] |
| electron-transfer-flavoprotein, beta polypeptide | NP_001976.1 | 6 | 31 | 0 | 12 | 24 | 1 | 0.02 | 1 | No |  |  |
| MAP3K12 binding inhibitory protein 1 | NP_057670.1 | 4 | 38 | 0 | 10 | 32 | 1 | 0.02 | 1 | Likely | Negative regulator of MUK/dual leucine zipper-bearing kinase. No direct data but MAPK pathway has a major role in cancer and apoptosis. | [171]  [172] |
| beta-tubulin cofactor C | NP_003183.1 | 5 | 33 | 0 | 11 | 26 | 1 | 0.021 | 1 | Possible | Molecular chaperone involved in tubulin folding and stimulation of GTPase activity. No specific data but cancer involvement seems plausible because of the role of tubulin. | [173] |
| insulin-like growth factor binding protein 1 | NP_000587.1 | 26 | 109 | 3 | 35 | 97 | 6 | 0.03 | 1 | Yes | Cell growth control, decreased in prostate cancer, increased level associated with rduced tumor growth. | [174] |
| lysophospholipase-like 1 | NP_620149.1 | 3 | 24 | 1 | 8 | 20 | 1 | 0.032 | 1 | Likely | No data on this particular gene but phospholipd metabolism is strongly perturbed in tumors and lysophospholipase 1 is strongly down-regulated in breast cancer. | [175] |
| Chorionic somatomammotropin hormone 2 isoform 2 | NP_072170.1 | 30 | 113 | 2 | 40 | 102 | 5 | 0.033 | 1 | Yes | Ectopic production of hormones is characteristic of many tumors, in some cases, they may have autocrine simulatory effect. | [176]  [177] |
| Williams Beuren syndrome chromosome region 20C isoform 1 | NP_115534 | 5 | 32 | 0 | 10 | 26 | 0 | 0.034 | 1 | Possible | Predicted rRNA/tRNA C5-methyltransferase; no specific data on this particular protein, however, the homologous NOL1 is a proliferating cell marker.. | [178] |

^a^The designations are as in Table 2.

**References**

1. Johnson ME, Viggiano L, Bailey JA, Abdul-Rauf M, Goodwin G, Rocchi M, Eichler EE: Positive selection of a gene family during the emergence of humans and African apes. *Nature* 2001, 413(6855):514-519.

2. Xiao J, Liu CC, Chen PL, Lee WH: RINT-1, a novel Rad50-interacting protein, participates in radiation-induced G(2)/M checkpoint control. *J Biol Chem* 2001, 276(9):6105-6111.

3. Timar J, Lapis K, Dudas J, Sebestyen A, Kopper L, Kovalszky I: Proteoglycans and tumor progression: Janus-faced molecules with contradictory functions in cancer. *Semin Cancer Biol* 2002, 12(3):173-186.

4. Sasisekharan R, Shriver Z, Venkataraman G, Narayanasami U: Roles of heparan-sulphate glycosaminoglycans in cancer. *Nat Rev Cancer* 2002, 2(7):521-528.

5. Filmus J, Selleck SB: Glypicans: proteoglycans with a surprise. *J Clin Invest* 2001, 108(4):497-501.

6. To-Figueras J, Gene M, Gomez-Catalan J, Pique E, Borrego N, Caballero M, Cruellas F, Raya A, Dicenta M, Corbella J: Microsomal epoxide hydrolase and glutathione S-transferase polymorphisms in relation to laryngeal carcinoma risk. *Cancer Lett* 2002, 187(1-2):95-101.

7. Ohashi Y, Kaneko SJ, Cupples TE, Young SR: Ubiquinol cytochrome c reductase (UQCRFS1) gene amplification in primary breast cancer core biopsy samples. *Gynecol Oncol* 2004, 93(1):54-58.

8. Modena P, Testi MA, Facchinetti F, Mezzanzanica D, Radice MT, Pilotti S, Sozzi G: UQCRH gene encoding mitochondrial Hinge protein is interrupted by a translocation in a soft-tissue sarcoma and epigenetically inactivated in some cancer cell lines. *Oncogene* 2003, 22(29):4586-4593.

9. Mendez J, Zou-Yang XH, Kim SY, Hidaka M, Tansey WP, Stillman B: Human origin recognition complex large subunit is degraded by ubiquitin-mediated proteolysis after initiation of DNA replication. *Mol Cell* 2002, 9(3):481-491.

10. Sun SY, Yue P, Wu GS, El-Deiry WS, Shroot B, Hong WK, Lotan R: Mechanisms of apoptosis induced by the synthetic retinoid CD437 in human non-small cell lung carcinoma cells. *Oncogene* 1999, 18(14):2357-2365.

11. Soprano DR, Qin P, Soprano KJ: Retinoic acid receptors and cancers. *Annu Rev Nutr* 2004, 24:201-221.

12. Pettersson F, Dalgleish AG, Bissonnette RP, Colston KW: Retinoids cause apoptosis in pancreatic cancer cells via activation of RAR-gamma and altered expression of Bcl-2/Bax. *Br J Cancer* 2002, 87(5):555-561.

13. Srinivas H, Juroske DM, Kalyankrishna S, Cody DD, Price RE, Xu XC, Narayanan R, Weigel NL, Kurie JM: c-Jun N-terminal kinase contributes to aberrant retinoid signaling in lung cancer cells by phosphorylating and inducing proteasomal degradation of retinoic acid receptor alpha. *Mol Cell Biol* 2005, 25(3):1054-1069.

14. Yang HS, Jansen AP, Komar AA, Zheng X, Merrick WC, Costes S, Lockett SJ, Sonenberg N, Colburn NH: The transformation suppressor Pdcd4 is a novel eukaryotic translation initiation factor 4A binding protein that inhibits translation. *Mol Cell Biol* 2003, 23(1):26-37.

15. Jansen AP, Camalier CE, Stark C, Colburn NH: Characterization of programmed cell death 4 in multiple human cancers reveals a novel enhancer of drug sensitivity. *Mol Cancer Ther* 2004, 3(2):103-110.

16. Sawai M, Yashiro M, Nishiguchi Y, Ohira M, Hirakawa K: Growth-inhibitory effects of the ketone body, monoacetoacetin, on human gastric cancer cells with succinyl-CoA: 3-oxoacid CoA-transferase (SCOT) deficiency. *Anticancer Res* 2004, 24(4):2213-2217.

17. Bell B, Scheer E, Tora L: Identification of hTAF(II)80 delta links apoptotic signaling pathways to transcription factor TFIID function. *Mol Cell* 2001, 8(3):591-600.

18. Berrieman HK, Lind MJ, Cawkwell L: Do beta-tubulin mutations have a role in resistance to chemotherapy? *Lancet Oncol* 2004, 5(3):158-164.

19. Antonyak MA, Miller AM, Jansen JM, Boehm JE, Balkman CE, Wakshlag JJ, Page RL, Cerione RA: Augmentation of tissue transglutaminase expression and activation by epidermal growth factor inhibit doxorubicin-induced apoptosis in human breast cancer cells. *J Biol Chem* 2004, 279(40):41461-41467.

20. Belkin AM, Zemskov EA, Hang J, Akimov SS, Sikora S, Strongin AY: Cell-surface-associated tissue transglutaminase is a target of MMP-2 proteolysis. *Biochemistry* 2004, 43(37):11760-11769.

21. Song SW, Fuller GN, Khan A, Kong S, Shen W, Taylor E, Ramdas L, Lang FF, Zhang W: IIp45, an insulin-like growth factor binding protein 2 (IGFBP-2) binding protein, antagonizes IGFBP-2 stimulation of glioma cell invasion. *Proc Natl Acad Sci U S A* 2003, 100(24):13970-13975.

22. Taanman JW, Kateeb I, Muntau AC, Jaksch M, Cohen N, Mandel H: A novel mutation in the deoxyguanosine kinase gene causing depletion of mitochondrial DNA. *Ann Neurol* 2002, 52(2):237-239.

23. Lotfi K, Mansson E, Peterson C, Eriksson S, Albertioni F: Low level of mitochondrial deoxyguanosine kinase is the dominant factor in acquired resistance to 9-beta-D-arabinofuranosylguanine cytotoxicity. *Biochem Biophys Res Commun* 2002, 293(5):1489-1496.

24. Pappas IS, Vizirianakis IS, Tsiftsoglou AS: Cloning, sequencing and expression of a cDNA encoding the mouse L35a ribosomal protein during differentiation of murine erythroleukemia (MEL) cells. *Cell Biol Int* 2001, 25(7):629-634.

25. Lopez CD, Martinovsky G, Naumovski L: Inhibition of cell death by ribosomal protein L35a. *Cancer Lett* 2002, 180(2):195-202.

26. Fox SB, Braganca J, Turley H, Campo L, Han C, Gatter KC, Bhattacharya S, Harris AL: CITED4 inhibits hypoxia-activated transcription in cancer cells, and its cytoplasmic location in breast cancer is associated with elevated expression of tumor cell hypoxia-inducible factor 1alpha. *Cancer Res* 2004, 64(17):6075-6081.

27. Kung AL, Zabludoff SD, France DS, Freedman SJ, Tanner EA, Vieira A, Cornell-Kennon S, Lee J, Wang B, Wang J, Memmert K, Naegeli HU, Petersen F, Eck MJ, Bair KW, Wood AW, Livingston DM: Small molecule blockade of transcriptional coactivation of the hypoxia-inducible factor pathway. *Cancer Cell* 2004, 6(1):33-43.

28. Kipreos ET, Lander LE, Wing JP, He WW, Hedgecock EM: cul-1 is required for cell cycle exit in C. elegans and identifies a novel gene family. *Cell* 1996, 85(6):829-839.

29. Pray TR, Parlati F, Huang J, Wong BR, Payan DG, Bennett MK, Issakani SD, Molineaux S, Demo SD: Cell cycle regulatory E3 ubiquitin ligases as anticancer targets. *Drug Resist Updat* 2002, 5(6):249-258.

30. Piva R, Liu J, Chiarle R, Podda A, Pagano M, Inghirami G: In vivo interference with Skp1 function leads to genetic instability and neoplastic transformation. *Mol Cell Biol* 2002, 22(23):8375-8387.

31. Gupta RA, Sarraf P, Mueller E, Brockman JA, Prusakiewicz JJ, Eng C, Willson TM, DuBois RN: Peroxisome proliferator-activated receptor gamma-mediated differentiation: a mutation in colon cancer cells reveals divergent and cell type-specific mechanisms. *J Biol Chem* 2003, 278(25):22669-22677.

32. Kawamata H, Fujimori T, Imai Y: TSC-22 (TGF-beta stimulated clone-22): a novel molecular target for differentiation-inducing therapy in salivary gland cancer. *Curr Cancer Drug Targets* 2004, 4(6):521-529.

33. Shostak KO, Dmitrenko VV, Garifulin OM, Rozumenko VD, Khomenko OV, Zozulya YA, Zehetner G, Kavsan VM: Downregulation of putative tumor suppressor gene TSC-22 in human brain tumors. *J Surg Oncol* 2003, 82(1):57-64.

34. Cosma MP, Pepe S, Annunziata I, Newbold RF, Grompe M, Parenti G, Ballabio A: The multiple sulfatase deficiency gene encodes an essential and limiting factor for the activity of sulfatases. *Cell* 2003, 113(4):445-456.

35. Reed MJ, Purohit A, Woo LW, Newman SP, Potter BV: Steroid sulfatase: molecular biology, regulation, and inhibition. *Endocr Rev* 2005, 26(2):171-202.

36. Zhang H, Kelly G, Zerillo C, Jaworski DM, Hockfield S: Expression of a cleaved brain-specific extracellular matrix protein mediates glioma cell invasion In vivo. *J Neurosci* 1998, 18(7):2370-2376.

37. Gary SC, Zerillo CA, Chiang VL, Gaw JU, Gray G, Hockfield S: cDNA cloning, chromosomal localization, and expression analysis of human BEHAB/brevican, a brain specific proteoglycan regulated during cortical development and in glioma. *Gene* 2000, 256(1-2):139-147.

38. Nutt CL, Matthews RT, Hockfield S: Glial tumor invasion: a role for the upregulation and cleavage of BEHAB/brevican. *Neuroscientist* 2001, 7(2):113-122.

39. Kominsky SL, Argani P, Korz D, Evron E, Raman V, Garrett E, Rein A, Sauter G, Kallioniemi OP, Sukumar S: Loss of the tight junction protein claudin-7 correlates with histological grade in both ductal carcinoma in situ and invasive ductal carcinoma of the breast. *Oncogene* 2003, 22(13):2021-2033.

40. Zheng JY, Yu D, Foroohar M, Ko E, Chan J, Kim N, Chiu R, Pang S: Regulation of the expression of the prostate-specific antigen by claudin-7. *J Membr Biol* 2003, 194(3):187-197.

41. Dong F, Gutkind JS, Larner AC: Granulocyte colony-stimulating factor induces ERK5 activation, which is differentially regulated by protein-tyrosine kinases and protein kinase C. Regulation of cell proliferation and survival. *J Biol Chem* 2001, 276(14):10811-10816.

42. Cameron SJ, Abe J, Malik S, Che W, Yang J: Differential role of MEK5alpha and MEK5beta in BMK1/ERK5 activation. *J Biol Chem* 2004, 279(2):1506-1512.

43. Lefebvre-Legendre L, Vaillier J, Benabdelhak H, Velours J, Slonimski PP, di Rago JP: Identification of a nuclear gene (FMC1) required for the assembly/stability of yeast mitochondrial F(1)-ATPase in heat stress conditions. *J Biol Chem* 2001, 276(9):6789-6796.

44. Werner SR, Lee PA, DeCamp MW, Crowell DN, Randall SK, Crowell PL: Enhanced cell cycle progression and down regulation of p21(Cip1/Waf1) by PRL tyrosine phosphatases. *Cancer Lett* 2003, 202(2):201-211.

45. Zeng Q, Dong JM, Guo K, Li J, Tan HX, Koh V, Pallen CJ, Manser E, Hong W: PRL-3 and PRL-1 promote cell migration, invasion, and metastasis. *Cancer Res* 2003, 63(11):2716-2722.

46. Jeong DG, Kim SJ, Kim JH, Son JH, Park MR, Lim SM, Yoon TS, Ryu SE: Trimeric structure of PRL-1 phosphatase reveals an active enzyme conformation and regulation mechanisms. *J Mol Biol* 2005, 345(2):401-413.

47. Gaggero A, De Ambrosis A, Mezzanzanica D, Piazza T, Rubartelli A, Figini M, Canevari S, Ferrini S: A novel isoform of pro-interleukin-18 expressed in ovarian tumors is resistant to caspase-1 and -4 processing. *Oncogene* 2004, 23(45):7552-7560.

48. Hitomi J, Katayama T, Eguchi Y, Kudo T, Taniguchi M, Koyama Y, Manabe T, Yamagishi S, Bando Y, Imaizumi K, Tsujimoto Y, Tohyama M: Involvement of caspase-4 in endoplasmic reticulum stress-induced apoptosis and Abeta-induced cell death. *J Cell Biol* 2004, 165(3):347-356.

49. Yang XF, Wu CJ, McLaughlin S, Chillemi A, Wang KS, Canning C, Alyea EP, Kantoff P, Soiffer RJ, Dranoff G, Ritz J: CML66, a broadly immunogenic tumor antigen, elicits a humoral immune response associated with remission of chronic myelogenous leukemia. *Proc Natl Acad Sci U S A* 2001, 98(13):7492-7497.

50. Cayla X, Van Hoof C, Bosch M, Waelkens E, Vandekerckhove J, Peeters B, Merlevede W, Goris J: Molecular cloning, expression, and characterization of PTPA, a protein that activates the tyrosyl phosphatase activity of protein phosphatase 2A. *J Biol Chem* 1994, 269(22):15668-15675.

51. McCright B, Rivers AM, Audlin S, Virshup DM: The B56 family of protein phosphatase 2A (PP2A) regulatory subunits encodes differentiation-induced phosphoproteins that target PP2A to both nucleus and cytoplasm. *J Biol Chem* 1996, 271(36):22081-22089.

52. Venanzoni MC, Giunta S, Muraro GB, Storari L, Crescini C, Mazzucchelli R, Montironi R, Seth A: Apolipoprotein E expression in localized prostate cancers. *Int J Oncol* 2003, 22(4):779-786.

53. Chen YC, Pohl G, Wang TL, Morin PJ, Risberg B, Kristensen GB, Yu A, Davidson B, Shih Ie M: Apolipoprotein E is required for cell proliferation and survival in ovarian cancer. *Cancer Res* 2005, 65(1):331-337.

54. Brambillasca F, Mosna G, Colombo M, Rivolta A, Caslini C, Minuzzo M, Giudici G, Mizzi L, Biondi A, Privitera E: Identification of a novel molecular partner of the E2A gene in childhood leukemia. *Leukemia* 1999, 13(3):369-375.

55. Irie Y, Yamagata K, Gan Y, Miyamoto K, Do E, Kuo CH, Taira E, Miki N: Molecular cloning and characterization of Amida, a novel protein which interacts with a neuron-specific immediate early gene product arc, contains novel nuclear localization signals, and causes cell death in cultured cells. *J Biol Chem* 2000, 275(4):2647-2653.

56. Lee SH, Shin MS, Kim HS, Lee HK, Park WS, Kim SY, Lee JH, Han SY, Park JY, Oh RR, Jang JJ, Han JY, Lee JY, Yoo NJ: Alterations of the DR5/TRAIL receptor 2 gene in non-small cell lung cancers. *Cancer Res* 1999, 59(22):5683-5686.

57. Pai SI, Wu GS, Ozoren N, Wu L, Jen J, Sidransky D, El-Deiry WS: Rare loss-of-function mutation of a death receptor gene in head and neck cancer. *Cancer Res* 1998, 58(16):3513-3518.

58. Shen C, Nathan C: Nonredundant antioxidant defense by multiple two-cysteine peroxiredoxins in human prostate cancer cells. *Mol Med* 2002, 8(2):95-102.

59. Noh DY, Ahn SJ, Lee RA, Kim SW, Park IA, Chae HZ: Overexpression of peroxiredoxin in human breast cancer. *Anticancer Res* 2001, 21(3B):2085-2090.

60. Foster DA, Xu L: Phospholipase D in cell proliferation and cancer. *Mol Cancer Res* 2003, 1(11):789-800.

61. Mahoney JA, Ntolosi B, DaSilva RP, Gordon S, McKnight AJ: Cloning and characterization of CPVL, a novel serine carboxypeptidase, from human macrophages. *Genomics* 2001, 72(3):243-251.

62. Joseph SB, Bradley MN, Castrillo A, Bruhn KW, Mak PA, Pei L, Hogenesch J, O'Connell R M, Cheng G, Saez E, Miller JF, Tontonoz P: LXR-dependent gene expression is important for macrophage survival and the innate immune response. *Cell* 2004, 119(2):299-309.

63. Joseph SB, Castrillo A, Laffitte BA, Mangelsdorf DJ, Tontonoz P: Reciprocal regulation of inflammation and lipid metabolism by liver X receptors. *Nat Med* 2003, 9(2):213-219.

64. Fukuchi J, Kokontis JM, Hiipakka RA, Chuu CP, Liao S: Antiproliferative effect of liver X receptor agonists on LNCaP human prostate cancer cells. *Cancer Res* 2004, 64(21):7686-7689.

65. Carreras MC, Franco MC, Peralta JG, Poderoso JJ: Nitric oxide, complex I, and the modulation of mitochondrial reactive species in biology and disease. *Mol Aspects Med* 2004, 25(1-2):125-139.

66. Gong TW, Huang L, Warner SJ, Lomax MI: Characterization of the human UBE3B gene: structure, expression, evolution, and alternative splicing. *Genomics* 2003, 82(2):143-152.

67. Townsend DM, Tew KD: The role of glutathione-S-transferase in anti-cancer drug resistance. *Oncogene* 2003, 22(47):7369-7375.

68. Suyama E, Kawasaki H, Nakajima M, Taira K: Identification of genes involved in cell invasion by using a library of randomized hybrid ribozymes. *Proc Natl Acad Sci U S A* 2003, 100(10):5616-5621.

69. Somlyo AV, Phelps C, Dipierro C, Eto M, Read P, Barrett M, Gibson JJ, Burnitz MC, Myers C, Somlyo AP: Rho kinase and matrix metalloproteinase inhibitors cooperate to inhibit angiogenesis and growth of human prostate cancer xenotransplants. *Faseb J* 2003, 17(2):223-234.

70. Yasuhara N, Takeda E, Inoue H, Kotera I, Yoneda Y: Importin alpha/beta-mediated nuclear protein import is regulated in a cell cycle-dependent manner. *Exp Cell Res* 2004, 297(1):285-293.

71. Wang Y, Decker SJ, Sebolt-Leopold J: Knockdown of Chk1, Wee1 and Myt1 by RNA interference abrogates G2 checkpoint and induces apoptosis. *Cancer Biol Ther* 2004, 3(3):305-313.

72. Okumura E, Fukuhara T, Yoshida H, Hanada Si S, Kozutsumi R, Mori M, Tachibana K, Kishimoto T: Akt inhibits Myt1 in the signalling pathway that leads to meiotic G2/M-phase transition. *Nat Cell Biol* 2002, 4(2):111-116.

73. Brown DM, Ruoslahti E: Metadherin, a cell surface protein in breast tumors that mediates lung metastasis. *Cancer Cell* 2004, 5(4):365-374.

74. Heizmann CW, Fritz G, Schafer BW: S100 proteins: structure, functions and pathology. *Front Biosci* 2002, 7:d1356-1368.

75. Harpio R, Einarsson R: S100 proteins as cancer biomarkers with focus on S100B in malignant melanoma. *Clin Biochem* 2004, 37(7):512-518.

76. Emberley ED, Murphy LC, Watson PH: S100A7 and the progression of breast cancer. *Breast Cancer Res* 2004, 6(4):153-159.

77. Yoshida A, Urasaki Y, Waltham M, Bergman AC, Pourquier P, Rothwell DG, Inuzuka M, Weinstein JN, Ueda T, Appella E, Hickson ID, Pommier Y: Human apurinic/apyrimidinic endonuclease (Ape1) and its N-terminal truncated form (AN34) are involved in DNA fragmentation during apoptosis. *J Biol Chem* 2003, 278(39):37768-37776.

78. Ito H, Matsuo K, Hamajima N, Mitsudomi T, Sugiura T, Saito T, Yasue T, Lee KM, Kang D, Yoo KY, Sato S, Ueda R, Tajima K: Gene-environment interactions between the smoking habit and polymorphisms in the DNA repair genes, APE1 Asp148Glu and XRCC1 Arg399Gln, in Japanese lung cancer risk. *Carcinogenesis* 2004, 25(8):1395-1401.

79. Bobola MS, Blank A, Berger MS, Stevens BA, Silber JR: Apurinic/apyrimidinic endonuclease activity is elevated in human adult gliomas. *Clin Cancer Res* 2001, 7(11):3510-3518.

80. Bates S, Rowan S, Vousden KH: Characterisation of human cyclin G1 and G2: DNA damage inducible genes. *Oncogene* 1996, 13(5):1103-1109.

81. Kim Y, Shintani S, Kohno Y, Zhang R, Wong DT: Cyclin G2 dysregulation in human oral cancer. *Cancer Res* 2004, 64(24):8980-8986.

82. Sharma S, Sommers JA, Driscoll HC, Uzdilla L, Wilson TM, Brosh RM, Jr.: The exonucleolytic and endonucleolytic cleavage activities of human exonuclease 1 are stimulated by an interaction with the carboxyl-terminal region of the Werner syndrome protein. *J Biol Chem* 2003, 278(26):23487-23496.

83. Jagmohan-Changur S, Poikonen T, Vilkki S, Launonen V, Wikman F, Orntoft TF, Moller P, Vasen H, Tops C, Kolodner RD, Mecklin JP, Jarvinen H, Bevan S, Houlston RS, Aaltonen LA, Fodde R, Wijnen J, Karhu A: EXO1 variants occur commonly in normal population: evidence against a role in hereditary nonpolyposis colorectal cancer. *Cancer Res* 2003, 63(1):154-158.

84. Thompson E, Meldrum CJ, Crooks R, McPhillips M, Thomas L, Spigelman AD, Scott RJ: Hereditary non-polyposis colorectal cancer and the role of hPMS2 and hEXO1 mutations. *Clin Genet* 2004, 65(3):215-225.

85. McGarvey T, Rosonina E, McCracken S, Li Q, Arnaout R, Mientjes E, Nickerson JA, Awrey D, Greenblatt J, Grosveld G, Blencowe BJ: The acute myeloid leukemia-associated protein, DEK, forms a splicing-dependent interaction with exon-product complexes. *J Cell Biol* 2000, 150(2):309-320.

86. Meissner M, Lopato S, Gotzmann J, Sauermann G, Barta A: Proto-oncoprotein TLS/FUS is associated to the nuclear matrix and complexed with splicing factors PTB, SRm160, and SR proteins. *Exp Cell Res* 2003, 283(2):184-195.

87. Kile BT, Schulman BA, Alexander WS, Nicola NA, Martin HM, Hilton DJ: The SOCS box: a tale of destruction and degradation. *Trends Biochem Sci* 2002, 27(5):235-241.

88. Sutherland KD, Lindeman GJ, Choong DY, Wittlin S, Brentzell L, Phillips W, Campbell IG, Visvader JE: Differential hypermethylation of SOCS genes in ovarian and breast carcinomas. *Oncogene* 2004, 23(46):7726-7733.

89. Stickeler E, Kittrell F, Medina D, Berget SM: Stage-specific changes in SR splicing factors and alternative splicing in mammary tumorigenesis. *Oncogene* 1999, 18(24):3574-3582.

90. Dominski Z, Yang XC, Kaygun H, Dadlez M, Marzluff WF: A 3' exonuclease that specifically interacts with the 3' end of histone mRNA. *Mol Cell* 2003, 12(2):295-305.

91. Kennedy S, Wang D, Ruvkun G: A conserved siRNA-degrading RNase negatively regulates RNA interference in C. elegans. *Nature* 2004, 427(6975):645-649.

92. Yeaman C, Ayala MI, Wright JR, Bard F, Bossard C, Ang A, Maeda Y, Seufferlein T, Mellman I, Nelson WJ, Malhotra V: Protein kinase D regulates basolateral membrane protein exit from trans-Golgi network. *Nat Cell Biol* 2004, 6(2):106-112.

93. Hurd C, Waldron RT, Rozengurt E: Protein kinase D complexes with C-Jun N-terminal kinase via activation loop phosphorylation and phosphorylates the C-Jun N-terminus. *Oncogene* 2002, 21(14):2154-2160.

94. Quevillon S, Robinson JC, Berthonneau E, Siatecka M, Mirande M: Macromolecular assemblage of aminoacyl-tRNA synthetases: identification of protein-protein interactions and characterization of a core protein. *J Mol Biol* 1999, 285(1):183-195.

95. Strelow A, Bernardo K, Adam-Klages S, Linke T, Sandhoff K, Kronke M, Adam D: Overexpression of acid ceramidase protects from tumor necrosis factor-induced cell death. *J Exp Med* 2000, 192(5):601-612.

96. Seelan RS, Qian C, Yokomizo A, Bostwick DG, Smith DI, Liu W: Human acid ceramidase is overexpressed but not mutated in prostate cancer. *Genes Chromosomes Cancer* 2000, 29(2):137-146.

97. Asano K, Kinzy TG, Merrick WC, Hershey JW: Conservation and diversity of eukaryotic translation initiation factor eIF3. *J Biol Chem* 1997, 272(2):1101-1109.

98. Saramaki O, Willi N, Bratt O, Gasser TC, Koivisto P, Nupponen NN, Bubendorf L, Visakorpi T: Amplification of EIF3S3 gene is associated with advanced stage in prostate cancer. *Am J Pathol* 2001, 159(6):2089-2094.

99. Marone R, Hess D, Dankort D, Muller WJ, Hynes NE, Badache A: Memo mediates ErbB2-driven cell motility. *Nat Cell Biol* 2004, 6(6):515-522.

100. Loeppen S, Schneider D, Gaunitz F, Gebhardt R, Kurek R, Buchmann A, Schwarz M: Overexpression of glutamine synthetase is associated with beta-catenin-mutations in mouse liver tumors during promotion of hepatocarcinogenesis by phenobarbital. *Cancer Res* 2002, 62(20):5685-5688.

101. Osada T, Sakamoto M, Nagawa H, Yamamoto J, Matsuno Y, Iwamatsu A, Muto T, Hirohashi S: Acquisition of glutamine synthetase expression in human hepatocarcinogenesis: relation to disease recurrence and possible regulation by ubiquitin-dependent proteolysis. *Cancer* 1999, 85(4):819-831.

102. Sato S, Tomomori-Sato C, Banks CA, Sorokina I, Parmely TJ, Kong SE, Jin J, Cai Y, Lane WS, Brower CS, Conaway RC, Conaway JW: Identification of mammalian Mediator subunits with similarities to yeast Mediator subunits Srb5, Srb6, Med11, and Rox3. *J Biol Chem* 2003, 278(17):15123-15127.

103. Boube M, Joulia L, Cribbs DL, Bourbon HM: Evidence for a mediator of RNA polymerase II transcriptional regulation conserved from yeast to man. *Cell* 2002, 110(2):143-151.

104. Yan J, Yu Y, Wang N, Chang Y, Ying H, Liu W, He J, Li S, Jiang W, Li Y, Liu H, Wang H, Xu Y: LFIRE-1/HFREP-1, a liver-specific gene, is frequently downregulated and has growth suppressor activity in hepatocellular carcinoma. *Oncogene* 2004, 23(10):1939-1949.

105. Hara H, Yoshimura H, Uchida S, Toyoda Y, Aoki M, Sakai Y, Morimoto S, Shiokawa K: Molecular cloning and functional expression analysis of a cDNA for human hepassocin, a liver-specific protein with hepatocyte mitogenic activity. *Biochim Biophys Acta* 2001, 1520(1):45-53.

106. Scheffer GL, Wijngaard PL, Flens MJ, Izquierdo MA, Slovak ML, Pinedo HM, Meijer CJ, Clevers HC, Scheper RJ: The drug resistance-related protein LRP is the human major vault protein. *Nat Med* 1995, 1(6):578-582.

107. Kickhoefer VA, Vasu SK, Rome LH: Vaults are the answer, what is the question? *Trends Cell Biol* 1996, 6(5):174-178.

108. Tessitore L, Marengo B, Vance DE, Papotti M, Mussa A, Daidone MG, Costa A: Expression of phosphatidylethanolamine N-methyltransferase in human hepatocellular carcinomas. *Oncology* 2003, 65(2):152-158.

109. Tessitore L, Dianzani I, Cui Z, Vance DE: Diminished expression of phosphatidylethanolamine N-methyltransferase 2 during hepatocarcinogenesis. *Biochem J* 1999, 337 ( Pt 1):23-27.

110. Tessitore L, Sesca E, Vance DE: Inactivation of phosphatidylethanolamine N-methyltransferase-2 in aflatoxin-induced liver cancer and partial reversion of the neoplastic phenotype by PEMT transfection of hepatoma cells. *Int J Cancer* 2000, 86(3):362-367.

111. Zaccheo O, Dinsdale D, Meacock PA, Glynn P: Neuropathy target esterase and its yeast homologue degrade phosphatidylcholine to glycerophosphocholine in living cells. *J Biol Chem* 2004, 279(23):24024-24033.

112. Kasai H, Nadano D, Hidaka E, Higuchi K, Kawakubo M, Sato TA, Nakayama J: Differential expression of ribosomal proteins in human normal and neoplastic colorectum. *J Histochem Cytochem* 2003, 51(5):567-574.

113. Welsh GI, Kadereit S, Coccia EM, Hovanessian AG, Meurs EF: Colocalization within the nucleolus of two highly related IFN-induced human nuclear phosphoproteins with nucleolin. *Exp Cell Res* 1999, 250(1):62-74.

114. Tagoh H, Kishi H, Muraguchi A: Molecular cloning and characterization of a novel stromal cell-derived cDNA encoding a protein that facilitates gene activation of recombination activating gene (RAG)-1 in human lymphoid progenitors. *Biochem Biophys Res Commun* 1996, 221(3):744-749.

115. Lykidis A, Jackson PD, Rock CO, Jackowski S: The role of CDP-diacylglycerol synthetase and phosphatidylinositol synthase activity levels in the regulation of cellular phosphatidylinositol content. *J Biol Chem* 1997, 272(52):33402-33409.

116. Baba Y, Tsukuda M, Mochimatsu I, Furukawa S, Kagata H, Nagashima Y, Koshika S, Imoto M, Kato Y: Cytostatic effect of inostamycin, an inhibitor of cytidine 5'-diphosphate 1,2-diacyl-sn-glycerol (CDP-DG): inositol transferase, on oral squamous cell carcinoma cell lines. *Cell Biol Int* 2001, 25(7):613-620.

117. Baba Y, Tsukuda M, Mochimatsu I, Furukawa S, Kagata H, Yoji, Nagashima, Sakai N, Koshika S, Imoto M, Kato Y: Inostamycin, an inhibitor of cytidine 5'-diphosphate 1,2-diacyl-sn-glycerol (CDP-DG): inositol transferase, suppresses invasion ability by reducing productions of matrix metalloproteinase-2 and -9 and cell motility in HSC-4 tongue carcinoma cell line. *Clin Exp Metastasis* 2000, 18(3):273-279.

118. Eichmuller S, Usener D, Dummer R, Stein A, Thiel D, Schadendorf D: Serological detection of cutaneous T-cell lymphoma-associated antigens. *Proc Natl Acad Sci U S A* 2001, 98(2):629-634.

119. Usener D, Schadendorf D, Koch J, Dubel S, Eichmuller S: cTAGE: a cutaneous T cell lymphoma associated antigen family with tumor-specific splicing. *J Invest Dermatol* 2003, 121(1):198-206.

120. Fossey SC, Kuroda S, Price JA, Pendleton JK, Freedman BI, Bowden DW: Identification and characterization of PRKCBP1, a candidate RACK-like protein. *Mamm Genome* 2000, 11(10):919-925.

121. Song HY, Dunbar JD, Zhang YX, Guo D, Donner DB: Identification of a protein with homology to hsp90 that binds the type 1 tumor necrosis factor receptor. *J Biol Chem* 1995, 270(8):3574-3581.

122. Masuda Y, Shima G, Aiuchi T, Horie M, Hori K, Nakajo S, Kajimoto S, Shibayama-Imazu T, Nakaya K: Involvement of tumor necrosis factor receptor-associated protein 1 (TRAP1) in apoptosis induced by beta-hydroxyisovalerylshikonin. *J Biol Chem* 2004, 279(41):42503-42515.

123. Maxwell PH: HIF-1's relationship to oxygen: simple yet sophisticated. *Cell Cycle* 2004, 3(2):156-159.

124. Conaway RC, Conaway JW: The von Hippel-Lindau tumor suppressor complex and regulation of hypoxia-inducible transcription. *Adv Cancer Res* 2002, 85:1-12.

125. Ivan M, Kaelin WG, Jr.: The von Hippel-Lindau tumor suppressor protein. *Curr Opin Genet Dev* 2001, 11(1):27-34.

126. Ichigotani Y, Fujii K, Hamaguchi M, Matsuda S: In search of a function for the E3B1/Abi2/Argbp1/NESH family (Review). *Int J Mol Med* 2002, 9(6):591-595.

127. Stradal T, Courtney KD, Rottner K, Hahne P, Small JV, Pendergast AM: The Abl interactor proteins localize to sites of actin polymerization at the tips of lamellipodia and filopodia. *Curr Biol* 2001, 11(11):891-895.

128. Zheng H, Ji C, Zou X, Wu M, Jin Z, Yin G, Li J, Feng C, Cheng H, Gu S, Xie Y, Mao Y: Molecular cloning and characterization of a novel human putative transmembrane protein homologous to mouse sideroflexin associated with sideroblastic anemia. *DNA Seq* 2003, 14(5):369-373.

129. Wu D, Govindasamy L, Lian W, Gu Y, Kukar T, Agbandje-McKenna M, McKenna R: Structure of human carnitine acetyltransferase. Molecular basis for fatty acyl transfer. *J Biol Chem* 2003, 278(15):13159-13165.

130. Komoto J, Yamada T, Takata Y, Konishi K, Ogawa H, Gomi T, Fujioka M, Takusagawa F: Catalytic mechanism of guanidinoacetate methyltransferase: crystal structures of guanidinoacetate methyltransferase ternary complexes. *Biochemistry* 2004, 43(45):14385-14394.

131. Roberts R, Sciorra VA, Morris AJ: Human type 2 phosphatidic acid phosphohydrolases. Substrate specificity of the type 2a, 2b, and 2c enzymes and cell surface activity of the 2a isoform. *J Biol Chem* 1998, 273(34):22059-22067.

132. Charroux B, Pellizzoni L, Perkinson RA, Yong J, Shevchenko A, Mann M, Dreyfuss G: Gemin4. A novel component of the SMN complex that is found in both gems and nucleoli. *J Cell Biol* 2000, 148(6):1177-1186.

133. Wan D, He M, Wang J, Qiu X, Zhou W, Luo Z, Chen J, Gu J: Two variants of the human hepatocellular carcinoma-associated HCAP1 gene and their effect on the growth of the human liver cancer cell line Hep3B. *Genes Chromosomes Cancer* 2004, 39(1):48-58.

134. Chadwick BP, Heath SK, Williamson J, Obermayr F, Patel L, Sheer D, Frischauf AM: The human homologue of the ninjurin gene maps to the candidate region of hereditary sensory neuropathy type I (HSNI). *Genomics* 1998, 47(1):58-63.

135. Araki T, Zimonjic DB, Popescu NC, Milbrandt J: Mechanism of homophilic binding mediated by ninjurin, a novel widely expressed adhesion molecule. *J Biol Chem* 1997, 272(34):21373-21380.

136. Marmorstein R: Structure and chemistry of the Sir2 family of NAD+-dependent histone/protein deactylases. *Biochem Soc Trans* 2004, 32(Pt 6):904-909.

137. Mutai H, Toyoshima Y, Sun W, Hattori N, Tanaka S, Shiota K: PAL31, a novel nuclear protein, expressed in the developing brain. *Biochem Biophys Res Commun* 2000, 274(2):427-433.

138. Santoro M, Carlomagno F, Melillo RM, Fusco A: Dysfunction of the RET receptor in human cancer. *Cell Mol Life Sci* 2004, 61(23):2954-2964.

139. Prowse AH, Vanderveer L, Milling SW, Pan ZZ, Dunbrack RL, Xu XX, Godwin AK: OVCA2 is downregulated and degraded during retinoid-induced apoptosis. *Int J Cancer* 2002, 99(2):185-192.

140. Hu P, Wu S, Sun Y, Yuan CC, Kobayashi R, Myers MP, Hernandez N: Characterization of human RNA polymerase III identifies orthologues for Saccharomyces cerevisiae RNA polymerase III subunits. *Mol Cell Biol* 2002, 22(22):8044-8055.

141. Eszlinger M, Holzapfel HP, Voigt C, Arkenau C, Paschke R: RGS 2 expression is regulated by TSH and inhibits TSH receptor signaling. *Eur J Endocrinol* 2004, 151(3):383-390.

142. Zhu Y, Hollmen J, Raty R, Aalto Y, Nagy B, Elonen E, Kere J, Mannila H, Franssila K, Knuutila S: Investigatory and analytical approaches to differential gene expression profiling in mantle cell lymphoma. *Br J Haematol* 2002, 119(4):905-915.

143. Tonjes A, Miedlich S, Holzapfel HP, Eszlinger M, Arkenau C, Paschke R: Expression of regulators of g protein signaling mRNA is differentially regulated in hot and cold thyroid nodules. *Thyroid* 2004, 14(11):896-901.

144. Ohhira M, Gasa S, Makita A, Sekiya C, Namiki M: Elevated carbohydrate phosphotransferase activity in human hepatoma and phosphorylation of cathepsin D. *Br J Cancer* 1991, 63(6):905-908.

145. Uehara Y, Gasa S, Makita A, Sakurada K, Miyazaki T: Increased N-acetylglucosamine-1-phosphotransferase activity in sera from patients with leukemia. *Jpn J Cancer Res* 1991, 82(1):82-85.

146. Wu X, Li H, Chen JD: The human homologue of the yeast DNA repair and TFIIH regulator MMS19 is an AF-1-specific coactivator of estrogen receptor. *J Biol Chem* 2001, 276(26):23962-23968.

147. Paolucci L, Rozengurt E: Protein kinase D in small cell lung cancer cells: rapid activation through protein kinase C. *Cancer Res* 1999, 59(3):572-577.

148. Thut CJ, Goodrich JA, Tjian R: Repression of p53-mediated transcription by MDM2: a dual mechanism. *Genes Dev* 1997, 11(15):1974-1986.

149. Zhu PP, Patterson A, Lavoie B, Stadler J, Shoeb M, Patel R, Blackstone C: Cellular localization, oligomerization, and membrane association of the hereditary spastic paraplegia 3A (SPG3A) protein atlastin. *J Biol Chem* 2003, 278(49):49063-49071.

150. Freeman JL, Gonzalo P, Pitcher JA, Claing A, Lavergne JP, Reboud JP, Lefkowitz RJ: Beta 2-adrenergic receptor stimulated, G protein-coupled receptor kinase 2 mediated, phosphorylation of ribosomal protein P2. *Biochemistry* 2002, 41(42):12850-12857.

151. Gardner-Thorpe J, Ito H, Ashley SW, Whang EE: Ribosomal protein P2: a potential molecular target for antisense therapy of human malignancies. *Anticancer Res* 2003, 23(6C):4549-4560.

152. Wang Y, Cheong D, Chan S, Hooi SC: Heparin/heparan sulfate interacting protein gene expression is up-regulated in human colorectal carcinoma and correlated with differentiation status and metastasis. *Cancer Res* 1999, 59(12):2989-2994.

153. Liu JJ, Zhang J, Ramanan S, Julian J, Carson DD, Hooi SC: Heparin/heparan sulfate interacting protein plays a role in apoptosis induced by anticancer drugs. *Carcinogenesis* 2004, 25(6):873-879.

154. Smyth MJ, Cretney E, Kershaw MH, Hayakawa Y: Cytokines in cancer immunity and immunotherapy. *Immunol Rev* 2004, 202:275-293.

155. Kioka N, Sakata S, Kawauchi T, Amachi T, Akiyama SK, Okazaki K, Yaen C, Yamada KM, Aota S: Vinexin: a novel vinculin-binding protein with multiple SH3 domains enhances actin cytoskeletal organization. *J Cell Biol* 1999, 144(1):59-69.

156. Mitsushima M, Suwa A, Amachi T, Ueda K, Kioka N: Extracellular signal-regulated kinase activated by epidermal growth factor and cell adhesion interacts with and phosphorylates vinexin. *J Biol Chem* 2004, 279(33):34570-34577.

157. Miinalainen IJ, Chen ZJ, Torkko JM, Pirila PL, Sormunen RT, Bergmann U, Qin YM, Hiltunen JK: Characterization of 2-enoyl thioester reductase from mammals. An ortholog of YBR026p/MRF1'p of the yeast mitochondrial fatty acid synthesis type II. *J Biol Chem* 2003, 278(22):20154-20161.

158. Moss DW, Raymond FD, Wile DB: Clinical and biological aspects of acid phosphatase. *Crit Rev Clin Lab Sci* 1995, 32(4):431-467.

159. Golabek AA, Kida E, Walus M, Kaczmarski W, Wujek P, Wisniewski KE: CLN3 disease process: missense point mutations and protein depletion in vitro. *Eur J Paediatr Neurol* 2001, 5 Suppl A:81-88.

160. Rylova SN, Amalfitano A, Persaud-Sawin DA, Guo WX, Chang J, Jansen PJ, Proia AD, Boustany RM: The CLN3 gene is a novel molecular target for cancer drug discovery. *Cancer Res* 2002, 62(3):801-808.

161. Izumi H, Torigoe T, Ishiguchi H, Uramoto H, Yoshida Y, Tanabe M, Ise T, Murakami T, Yoshida T, Nomoto M, Kohno K: Cellular pH regulators: potentially promising molecular targets for cancer chemotherapy. *Cancer Treat Rev* 2003, 29(6):541-549.

162. Cramer P, Bushnell DA, Fu J, Gnatt AL, Maier-Davis B, Thompson NE, Burgess RR, Edwards AM, David PR, Kornberg RD: Architecture of RNA polymerase II and implications for the transcription mechanism. *Science* 2000, 288(5466):640-649.

163. Teng MS, Stephens R, Du Pasquier L, Freeman T, Lindquist JA, Trowsdale J: A human TAPBP (TAPASIN)-related gene, TAPBP-R. *Eur J Immunol* 2002, 32(4):1059-1068.

164. Ostergaard M, Rasmussen HH, Nielsen HV, Vorum H, Orntoft TF, Wolf H, Celis JE: Proteome profiling of bladder squamous cell carcinomas: identification of markers that define their degree of differentiation. *Cancer Res* 1997, 57(18):4111-4117.

165. Westendorf JM, Konstantinov KN, Wormsley S, Shu MD, Matsumoto-Taniura N, Pirollet F, Klier FG, Gerace L, Baserga SJ: M phase phosphoprotein 10 is a human U3 small nucleolar ribonucleoprotein component. *Mol Biol Cell* 1998, 9(2):437-449.

166. Goldstein AL, Badamchian M: Thymosins: chemistry and biological properties in health and disease. *Expert Opin Biol Ther* 2004, 4(4):559-573.

167. Takai T, Nakamura A, Akiyama K: Fc receptors as potential targets for the treatment of allergy, autoimmune disease and cancer. *Curr Drug Targets Immune Endocr Metabol Disord* 2003, 3(3):187-197.

168. Peipp M, Valerius T: Bispecific antibodies targeting cancer cells. *Biochem Soc Trans* 2002, 30(4):507-511.

169. O'Toole PJ, Inoue T, Emerson L, Morrison IE, Mackie AR, Cherry RJ, Norton JD: Id proteins negatively regulate basic helix-loop-helix transcription factor function by disrupting subnuclear compartmentalization. *J Biol Chem* 2003, 278(46):45770-45776.

170. Sakurai D, Tsuchiya N, Yamaguchi A, Okaji Y, Tsuno NH, Kobata T, Takahashi K, Tokunaga K: Crucial role of inhibitor of DNA binding/differentiation in the vascular endothelial growth factor-induced activation and angiogenic processes of human endothelial cells. *J Immunol* 2004, 173(9):5801-5809.

171. Fukuyama K, Yoshida M, Yamashita A, Deyama T, Baba M, Suzuki A, Mohri H, Ikezawa Z, Nakajima H, Hirai S, Ohno S: MAPK upstream kinase (MUK)-binding inhibitory protein, a negative regulator of MUK/dual leucine zipper-bearing kinase/leucine zipper protein kinase. *J Biol Chem* 2000, 275(28):21247-21254.

172. Freeman SM, Whartenby KA: The role of the mitogen-activated protein kinase cellular signaling pathway in tumor cell survival and apoptosis. *Drug News Perspect* 2004, 17(4):237-242.

173. Tian G, Huang Y, Rommelaere H, Vandekerckhove J, Ampe C, Cowan NJ: Pathway leading to correctly folded beta-tubulin. *Cell* 1996, 86(2):287-296.

174. Tennant MK, Vessella RL, Sprenger CC, Sikes RA, Hwa V, Baker LD, Plymate SR: Insulin-like growth factor binding protein-related protein 1 (IGFBP-rP1/mac 25) is reduced in human prostate cancer and is inversely related to tumor volume and proliferation index in Lucap 23.12 xenografts. *Prostate* 2003, 56(2):115-122.

175. Glunde K, Jie C, Bhujwalla ZM: Molecular causes of the aberrant choline phospholipid metabolism in breast cancer. *Cancer Res* 2004, 64(12):4270-4276.

176. DeLellis RA, Xia L: Paraneoplastic endocrine syndromes: a review. *Endocr Pathol* 2003, 14(4):303-317.

177. Braunstein GD: Placental proteins as tumor markers. *Immunol Ser* 1990, 53:673-701.

178. Trere D, Migaldi M, Montanaro L, Pession A, Derenzini M: p120 expression provides a reliable indication of the rapidity of cell duplication in cancer cells independently of tumour origin. *J Pathol* 2000, 192(2):216-220.
